# Capsular Sialyltransferase Specificity Mediates Different Phenotypes in *Streptococcus suis* and Group B *Streptococcus*

**DOI:** 10.3389/fmicb.2018.00545

**Published:** 2018-04-03

**Authors:** David Roy, Daisuke Takamatsu, Masatoshi Okura, Guillaume Goyette-Desjardins, Marie-Rose Van Calsteren, Audrey Dumesnil, Marcelo Gottschalk, Mariela Segura

**Affiliations:** ^1^Faculty of Veterinary Medicine, Swine and Poultry Infectious Disease Research Centre, University of Montreal, Saint-Hyacinthe, QC, Canada; ^2^Division of Bacterial and Parasitic Diseases, National Institute of Animal Health, National Agriculture and Food Research Organization, Tsukuba, Japan; ^3^The United Graduate School of Veterinary Sciences, Gifu University, Gifu, Japan; ^4^Saint-Hyacinthe Research and Development Centre, Agriculture and Agri-Food Canada, Saint-Hyacinthe, QC, Canada

**Keywords:** *Streptococcus suis*, Group B *Streptococcus*, capsular polysaccharide, sialyltransferase, α-2, 6 sialic acid, α-2, 3 sialic acid

## Abstract

The capsular polysaccharide (CPS) represents a key virulence factor for most encapsulated streptococci. *Streptococcus suis* and Group B *Streptococcus* (GBS) are both well-encapsulated pathogens of clinical importance in veterinary and/or human medicine and responsible for invasive systemic diseases. *S. suis* and GBS are the only Gram-positive bacteria which express a sialylated CPS at their surface. An important difference between these two sialylated CPSs is the linkage between the side-chain terminal galactose and sialic acid, being α-2,6 for *S. suis* but α-2,3 for GBS. It is still unclear how sialic acid may affect CPS production and, consequently, the pathogenesis of the disease caused by these two bacterial pathogens. Here, we investigated the role of sialic acid and the putative effect of sialic acid linkage modification in CPS synthesis using inter-species allelic exchange mutagenesis. To this aim, a new molecular biogenetic approach to express CPS with modified sialic acid linkage was developed. We showed that sialic acid (and its α-2,6 linkage) is crucial for *S. suis* CPS synthesis, whereas for GBS, CPS synthesis may occur in presence of an α-2,6 sialyltransferase or in absence of sialic acid moiety. To evaluate the effect of the CPS composition/structure on sialyltransferase activity, two distinct capsular serotypes within each bacterial species were compared (*S. suis* serotypes 2 and 14 and GBS serotypes III and V). It was demonstrated that the observed differences in sialyltransferase activity and specificity between *S. suis* and GBS were serotype unrestricted. This is the first time that a study investigates the interspecies exchange of capsular sialyltransferase genes in Gram-positive bacteria. The obtained mutants represent novel tools that could be used to further investigate the immunomodulatory properties of sialylated CPSs. Finally, in spite of common CPS structural characteristics and similarities in the *cps* loci, sialic acid exerts differential control of CPS expression by *S. suis* and GBS.

## Introduction

Capsular polysaccharides (CPSs) play critical roles in the pathogenesis of the disease caused by several bacterial pathogens, including streptococci. Indeed, the CPS expressed at the bacterial surface is one of the primary structures that interacts with host cells during colonization—the first step of the infection—and, more importantly, during invasion and dissemination within the host. *Streptococcus suis* and Group B *Streptococcus* (GBS; also known as *Streptococcus agalactiae*) are two well-encapsulated Gram-positive bacteria that were extensively studied in the last years/decades due to their veterinary and/or medical significance. Variations in CPS antigenicity allow these two bacterial species classification into serotypes, which differ in their clinical importance and epidemiological features, including geographical distribution (Cieslewicz et al., 2005; Johri et al., 2006; Goyette-Desjardins et al., 2014).

*S. suis* is a zoonotic pathogen that causes severe economic problems in swine production and represents a serious risk for public health. The most common clinical outcomes caused by *S. suis* in pigs are meningitis and septicemia with sudden death. In humans, meningitis and severe streptococcal toxic shock-like syndrome is also frequently reported, especially in Asian countries. Other pathologies include arthritis, endocarditis, and pneumonia. Of the initially described 35 capsular types or serotypes, *S. suis* type 2 predominates worldwide in both pigs and humans. Besides this important and highly virulent serotype, type 14 is also emerging as a threat to human health (Goyette-Desjardins et al., 2014). On the other hand, GBS is an important cause of severe invasive bacterial infections in humans worldwide (Johri et al., 2006; Madzivhandila et al., 2011). Clinical manifestations of GBS infection include pneumonia, septicemia, and meningitis in newborns and infants. GBS diseases also occur in pregnant women and have been recognized as an emerging cause of life-threatening invasive infections in adults, particularly the elderly and immunocompromised patients. To date, GBS is classified into 10 different serotypes, and type III is the most common type in GBS meningitis, whereas serotype V has long been recognized as a leading cause of invasive disease in adults (Johri et al., 2006; Madzivhandila et al., 2011).

Besides the common feature of being encapsulated and inducing similar pathologies*, S. suis* and GBS both use the Wzx/Wzy-dependent pathway to express their CPSs (Cieslewicz et al., 2005; Okura et al., 2013). The Wzx/Wzy-dependent pathway is characterized by the implication of two key enzymes: the Wzy polymerase and the Wzx flippase. The CPS structures of GBS types III and V (Wessels et al., 1987, 1991; Wessels and Kasper, 1990) and of *S. suis* types 2 and 14 (Van Calsteren et al., 2010, 2013) have already been determined. Although *S. suis* types 2 and 14 and GBS types III and V share common CPS structural elements, the CPS plays different roles in the pathogenesis of the disease. Indeed, it was demonstrated that the *S. suis* CPS is a critical antiphagocytic factor that protects bacteria against phagocytosis by macrophages, dendritic cells, and neutrophils (Charland et al., 1998; Chabot-Roy et al., 2006; Lecours et al., 2011a,b). The *S. suis* CPS is thus considered as a shielding factor that allows bacterial evasion of immunoclearance and characterizes *S. suis* as a strictly extracellular pathogen. In contrast, GBS is easily internalized at high numbers by dendritic cells and macrophages in spite of a thick CPS, being able to survive intracellularly for a transient period of time (Segura et al., 1998; Segura, 2012; Lemire et al., 2014).

**Figure 1:**
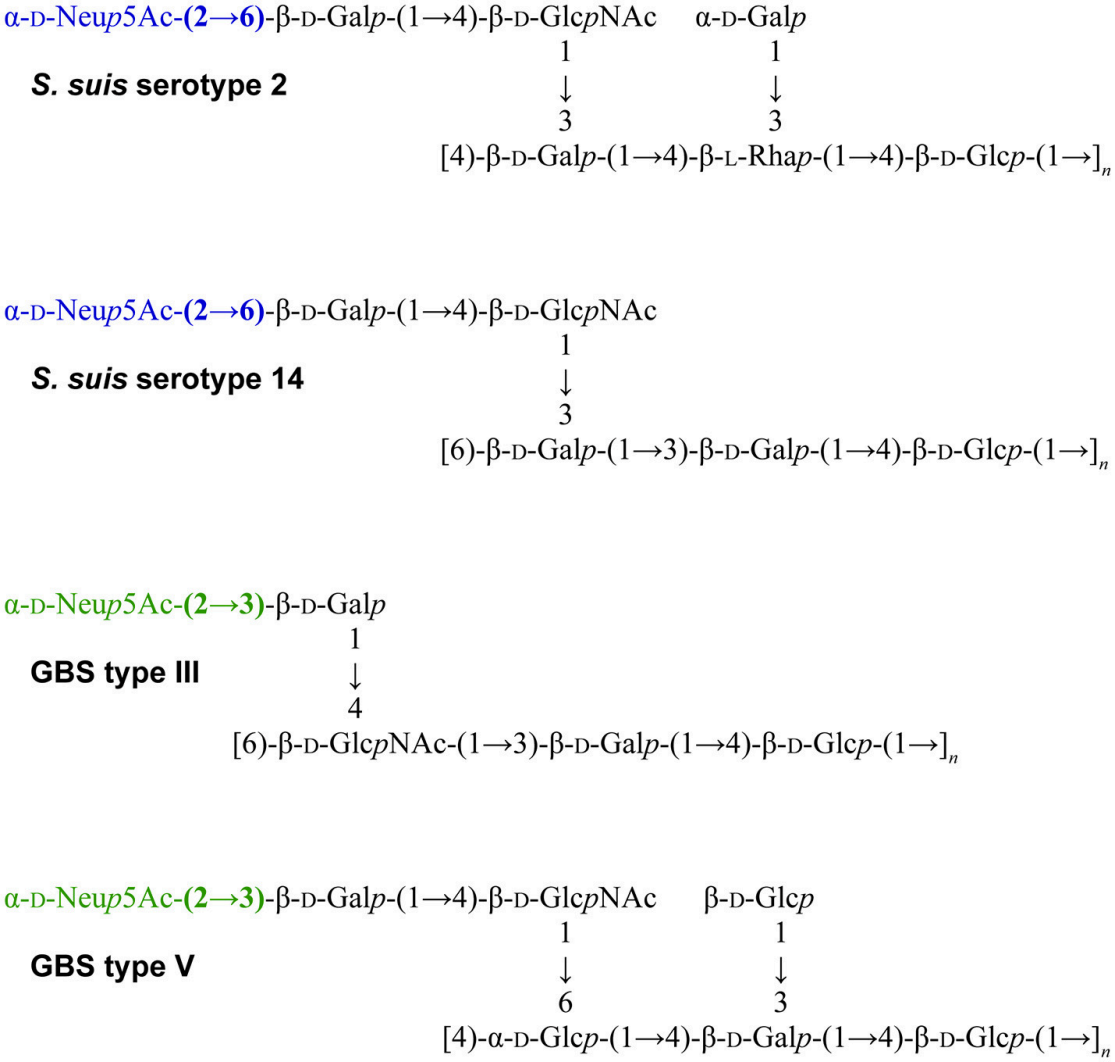
Reported structures for the capsular polysaccharide repeating units of *S. suis* serotypes 2 (Van Calsteren et al., 2010) and 14 (Van Calsteren et al., 2013) and of Group B *Streptococcus* (GBS) types III (Wessels et al., 1987; Wessels and Kasper, 1990) and V (Wessels et al., 1991).

Another striking feature of these two pathogens is that *S. suis* and GBS are the only Gram-positive bacteria expressing a sialylated CPS. Interestingly, there is a difference in the linkage between the side-chain terminal galactose and sialic acid. Indeed, *S. suis* expresses sialic acid α-2,6-linked to the adjacent galactose rather than an α-2,3-linked sialic acid as is the case for GBS (Van Calsteren et al., 2010, 2013; Okura et al., 2013; Figure 1). It was hypothesized that the type of sialic acid linkage may differently modulate immune cell activation and, consequently, may have an impact on bacterial-host interactions (Bax et al., 2011). Yet, this remains to be investigated in the context of *S. suis* and GBS infections.

In this study, we firstly evaluated the role of sialic acid in the synthesis and export of CPS by Gram-positive bacteria by deletion of genes encoding the sialyltransferases or those involved in the sialic acid synthesis pathway. Secondly, to specifically study the role of sialic acid linkage in *S. suis* (α-2,6) and GBS (α-2,3), we constructed *S. suis* type 2 or type 14 substitution mutants possessing the GBS type III α-2,3-sialyltransferase instead of the native α-2,6-sialyltransferase. Conversely, we constructed GBS type III and V mutants possessing the exogenous *S. suis* α-2,6-sialyltransferase. Using this novel genetic approach, we demonstrated a critical role of not only the presence of sialic acid, but more importantly its type of linkage in *S. suis* CPS production by two distinct serotypes. In contrast, GBS was still able to express asialo CPS or α-2,6-linked sialylated CPS irrespectively of the serotype. This is the first time that a study investigates the interspecies exchange of capsular sialyltransferase genes in Gram-positive bacteria.

## Materials and methods

### Plasmids, bacterial strains, and culture conditions

The well-encapsulated virulent *S. suis* serotype 2 strain P1/7, *S. suis* serotype 14 strain DAN13730, GBS type III strain COH1, and GBS type V strain CJB111 (ATCC BAA-23) were used as the host (wild-type) strains for in-frame allelic deletion mutagenesis. Bacterial strains and plasmids used in this study are listed and described in Table 1. Streptococcal strains were grown in Todd-Hewitt broth (THB) or agar (THA) (Becton-Dickinson, Sparks, MD) at 37°C. *Escherichia coli* strains were grown in Luria-Bertani broth or agar (Becton-Dickinson) at 37°C. When needed, antibiotics (Sigma, Oakville, ON, Canada) were added to the culture media at the following concentrations: for *S. suis*, spectinomycin (Sp) at 100 μg/ml; for *E. coli*, kanamycin and Sp at 50 μg/ml, and ampicillin at 100 μg/ml. Experiments were carried out in a BSL-2 certified laboratory.

**Table 1:** Bacterial strains and plasmids used in this study.

**Strain/Plasmid**	**General Characteristics**	**Source/References**
***Escherichia coli***		
TOP 10	F-*mrcA* Δ*(mrr-hsd*RMS*-mcr*BC*)φ80 lacZ*Δ*M5* Δ*lac*X74 *rec*A1 *ara*D139 Δ(*ara-leu*) 7697 *gal*U *gal*K *rps*L (StrR) *end*A1 *nup*G	Invitrogen
MC1061	*araD139 Δ*(*ara-leu*)*7697 Δlac*X74 *galU galK hsdR2*(rK-mK+) *mcrB1 rpsL*	Casadaban and Cohen, 1980
***Streptococcus suis*** **(SS) serotype 2**		
SS2 WT	P1/7 wild type (WT), highly encapsulated serotype 2 strain isolated from a clinical case of swine infection in the United Kingdom	Slater et al., 2003
SS2Δ*cps*	Non-encapsulated isogenic mutant strain derived from strain P1/7. In-frame deletion of the *cps2F* gene (rhamnosyltransferase; locus tag: SSU0520)	Lecours et al., 2011a
SS2ΔsiaT	Isogenic mutant strain derived from strain P1/7. Indirect in-frame deletion of the *cps2N* gene (sialyltransferase; locus tag: SSU0533)	This work
SS2compΔsiaT	Mutant SS2ΔsiaT complemented with pMX1*cps2N*	This work
SS2sia2,3	Isogenic mutant strain derived from strain P1/7. Indirect substitution of the *cps2N* gene by the *cps3K* gene of GBS type III (sialyltransferase; locus tag: GBSCOH1_1070)	This work
SS2Δsynth	Non-encapsulated isogenic mutant strain derived from strain P1/7. In-frame deletion of the *neu2C* gene (UDP-*N*-acetylglucosamine 2-epimerase; locus tag: SSU0536)	Lecours et al., 2011a
SS2Δsynth/ΔsiaT	Isogenic mutant strain derived from strain P1/7. In-frame deletions of the *neu2C* and *cps2N* genes	This work
SS2Δsynth/sia2,3	Isogenic mutant strain derived from strain P1/7. In-frame deletion of the *neu2C* gene and substitution of the *cps2N* gene by the *cps3K* gene of GBS type III	This work
***Streptococcus suis*** **(SS) serotype 14**		
SS14 WT	DAN13730 wild type (WT), highly encapsulated serotype 14 strain isolated from a human case in the Netherlands	Gottschalk et al., 1989
SS14Δ*cps*	Non-encapsulated isogenic mutant strain derived from strain DAN13730. In-frame deletion of the *cps14B* gene (chain length determinant protein Wzd; locus tag: equivalent of SSU0516 in P1/7)	Roy et al., 2015
SS14sia2,3	Isogenic mutant strain derived from strain DAN13730. Indirect substitution of the sialyltransferase *cps14N* gene (locus tag: equivalent of SSU0533 in P1/7) by the *cps3K* gene of GBS type III	This work
SS14Δsynth	Non-encapsulated isogenic mutant strain derived from strain DAN13730. In-frame deletion of the *neu14C* gene (UDP-*N*-acetylglucosamine 2-epimerase; locus tag: equivalent of SSU0536 in P1/7)	Roy et al., 2015
SS14Δsynth/sia2,3	Isogenic mutant strain derived from strain DAN13730. In-frame deletion of the *neu14C* gene and substitution of the *cps14N* gene by the *cps3K* gene of GBS type III	This work
**Group B** ***Streptococcus*** **(GBS) serotype III**		
GBSIII WT	COH1 wild type (WT), well-encapsulated type III strain isolated from an infant with sepsis and meningitis	Chaffin et al., 2005
GBSIIIsia2,6	Isogenic mutant strain derived from strain COH1. Direct substitution of sialyltransferase *cps3K* gene by *cps2N* gene of *S. suis* type 2	This work
GBSIIIΔ*cps*	Non-encapsulated isogenic mutant strain derived from strain COH1. In-frame deletion of the *cpsE* gene (glucosyltransferase; locus tag: GBSCOH1_1075)	Lecours et al., 2012
GBSIIIΔsynth	Intermediate-encapsulated isogenic mutant strain derived from strain COH1. In-frame deletion of the *neu3B* gene (sialic acid synthase; locus tag: GBSCOH1_1066)	Lecours et al., 2012
**Group B** ***Streptococcus*** **(GBS) serotype V**		
GBSV WT	CJB111 wild type (WT), well-encapsulated type V strain isolated from neonate with septicemia	ATCC
GBSVsia2,6	Isogenic mutant strain derived from strain CJB111. Direct substitution of *cps5K* gene (sialyltransferase; locus tag: SAG1163) by *cps2N* gene of *S. suis* type 2	This work
GBSVΔsynth	Isogenic mutant strain derived from strain CJB111. In-frame deletion of the *neu5B* gene (sialic acid synthase; locus tag: SAG1161)	This work
GBSVΔsiaT	Isogenic mutant strain derived from strain CJB111. In-frame deletion of the *cps5K* gene	This work
GBSVΔ*cps*	Non-encapsulated isogenic mutant strain derived from strain CJB111. In-frame deletion of the *cpsE* gene (glucosyltransferase; locus tag: SAG1171)	Lemire et al., 2014
**Plasmids**		
pCR2.1	Ap^r^, Km^r^, *oriR*(f1) MCS *oriR* (ColE1)	Invitrogen
pSET4s	Thermosensitive vector for allelic replacement. Replication functions of pG+host3, MCS *oriR* pUC19 *lacZ* Sp^R^	Takamatsu et al., 2001b
pMX1	Replication functions of pSSU1, MCS pUC19 lacZ Sp^R^, malQ promoter of *S. suis*, derivative of pSET2	Takamatsu et al., 2001b
p4Δ*neuC*	pSET4s carrying the construct for *neuC* allelic replacement (*S. suis* serotypes 2 and 14)	This work
p4Δ*cps2N*	pSET4s carrying the construct for *cps2N* indirect allelic replacement	This work
p4*neuC*	pSET4s carrying intact *neuC* gene for *neuC* reintroduction (*S. suis* serotypes 2 and 14)	This work
p4sia2,3_2	pSET4s carrying construct for allelic replacement of *S. suis cps2N* gene by *cps3K* from GBS type III	This work
p4sia2,3_14	pSET4s carrying construct for allelic replacement of *S. suis cps14N* gene by *cps3K* from GBS type III	This work
p4sia2,6_III	pSET-4s carrying construct for allelic replacement of GBS type III *cps3K* gene by *cps2N* from *S. suis* type 2	This work
p4sia2,6_V	pSET4s carrying construct for allelic replacement of GBS type V *cps5K* gene by *cps2N* from *S. suis* type 2	This work
p4Δ*neu5B*	pSET4s carrying construct for allelic deletion of GBS type V *neu5B* gene	This work
p4Δ*cps5K*	pSET4s carrying construct for allelic deletion of GBS type V *cps5K* gene	This work
pMX1*cps2N*	pMX1 complementation vector carrying intact *cps2N* gene	This work

### DNA manipulations

*S. suis* genomic DNA was purified by InstaGene Matrix solution (BioRad Laboratories, Hercules, CA). Accession numbers for reference *cps* loci sequences are: *S. suis* serotype 2: BR001000; *S. suis* serotype 14: AB737822; GBS type III: HG939456.1; and GBS type V: NC_004116. Transformations of *E. coli* were performed as recommended by the manufacturer (Invitrogen, Burlington, ON, Canada). Extraction and purification of recombinant plasmids were performed with QIAprep Spin Miniprep kit (Qiagen, Valencia, CA). Restriction enzymes were purchased from TaKaRa Bio (Otsu, Shiga, Japan) and used according to the manufacturer's directions. Alkaline phosphatase for plasmid dephosphorylation was purchased from MP Biomedicals (Solon, OH). PCR reactions were carried out with the iProof proofreading DNA polymerase (BioRad Laboratories) or with Taq DNA polymerase (Qiagen). Oligonucleotide primers were from IDT (Coralville, IA) and are listed in Supplementary Table S1. Amplification products were purified with the QIAgen PCR purification kit (Qiagen) and sequenced with an ABI 310 automated DNA sequencer using the ABI PRISM dye terminator cycle sequencing kit (Applied Biosystems, Foster City, CA).

### RT-PCR analysis of the *S. suis cps* locus

Bacterial RNA from serotype 2 strain P1/7 overnight culture was extracted with TRIZOL reagent (Invitrogen) as recommended by the manufacturer. RNase-free DNase (BioRad Laboratories) was used to treat RNA samples to remove contaminant genomic DNA. Reverse-transcriptase experiments were carried out with Qiagen One Step RT-PCR kit following the manufacturer's protocol. Primers used for RT-PCR experiments (Supplementary Table S1) were designed for each gene within the *cps* locus based on the available *S. suis* serotype 2 *cps* locus sequence (Accession # BR001000). PCR amplification from cDNA was carried out with NEB Taq polymerase (NEB, Ipswich, MA) with the following specific cycling conditions: 94°C for 5 min followed by 40 cycles at 94°C for 30 s, 47°C for 30 s, and 72°C for 1 min/kb. RNA samples without reverse-transcription step were used as templates to verify absence of genomic DNA in the samples.

### Indirect deletion of the sialyltransferase gene (*cps2N*) in *S. suis* serotype 2

**Figure 2:**
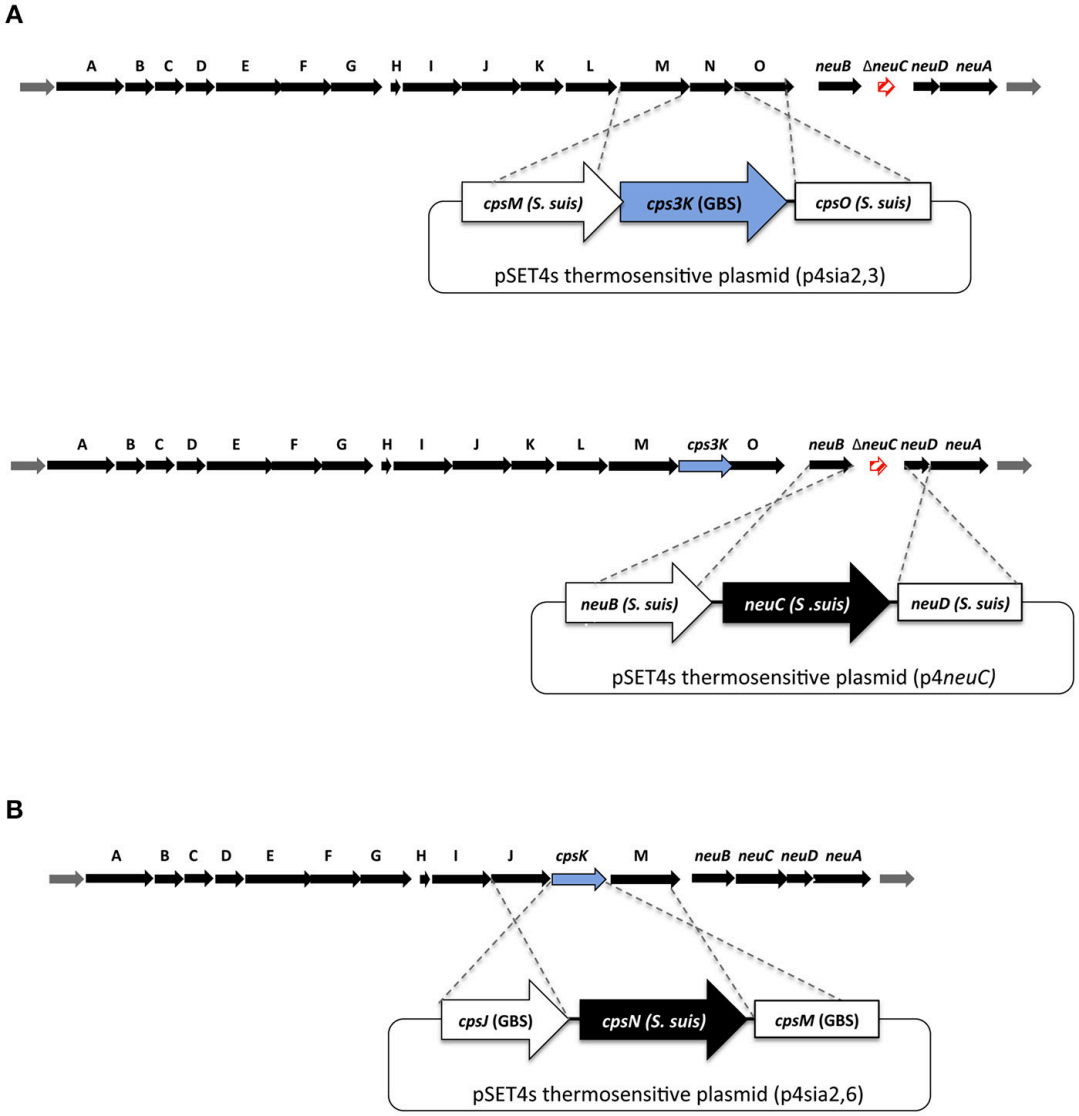
Schematic representation of mutagenesis procedures to obtain sialyltransferase substitution mutants in **(A)** *S. suis* and **(B)** Group B *Streptococcus* (GBS). (**A**; top part) Non-lethal mutation in *neuC* (Δsynth) was used first to abolish capsular polysaccharide (CPS) production. Gene substitution plasmids p4sia2,3_2 and p4sia2,3_14 were introduced into *S. suis* SS2Δsynth (serotype 2) and SS14Δsynth (serotype 14) mutants, respectively. (**A**; bottom part) In order to reintroduce a functional *neuC* gene into SS2Δ*neu2C*/*cps3K* and SS14Δ*neu14C*/*cps3K* mutants, the p4*neuC* plasmid was introduced into SS2Δ*neu2C*/*cps3K* and SS14Δ*neu14C*/*cps3K* mutants to obtain indirect substitution mutants SS2sia2,3 and SS14sia2,3. **(B)** In order to substitute GBS type III and V sialyltransferases, direct gene replacement by double-crossover homologous recombination system was used by introducing mutation plasmids p4sia2,6_III and p4sia2,6_V.

In order to inhibit CPS production and thus by-pass the lethality induced by mutation of the sialyltransferase (*cps2N*) gene in *S. suis* (Lakkitjaroen et al., 2014), we developed a “three-step” approach based on gene deletion/insertion by double cross-over homologous recombination to delete the *cps2N* sialyltransferase gene in *S. suis* (Figure 2).

First, precise in-frame deletion in the *neu2C* gene was achieved using splicing-by-overlap-extension PCR (Warrens et al., 1997) as previously described, to generate the SS2Δsynth (Δ*neu2C*) non-encapsulated mutant. The *neuC* mutation was previously shown to be non-lethal and was already successfully complemented (Lecours et al., 2012; Roy et al., 2015). Briefly, overlapping PCR products generated by PCR were cloned into the pCR2.1 TA-cloning vector (Invitrogen), extracted using restriction-enzyme digestion, and cloned into the thermosensitive *E. coli-S. suis* shuttle vector pSET4s, giving rise to the p4Δ*neuC* mutation vector. Final constructions of the pSET4s vector were electroporated into *S. suis* competent cells with a Biorad Gene Pulser Xcell apparatus (BioRad Laboratories) under specific conditions (12.5 kV/cm, 200 Ω, and 25 μF), and cells were plated on THA supplemented with Sp (THA+SP) and incubated for 3 days at 28°C. Several Sp-resistant colonies were then subcultured again on THA+SP for 3 days at 28°C. Next, the candidates were cultured on THA+SP and incubated at 37°C for two successive passages and then screened for first crossing-over event. Loss of vector was induced by incubation of candidates at 28°C. Temperature and Sp-resistant clones were successively cultured on THA and THA+SP to obtain Sp-sensitive candidates. Deletion of the gene was confirmed by PCR and sequence analysis.

Next, to knockdown *cps2N* and obtain the double mutant SS2Δsynth/ΔsiaT *(*Δ*neu2C*/Δ*cps2N*), the plasmid p4Δ*cps2N* was also constructed by using splicing-by-overlap-extension PCR as described above (Warrens et al., 1997). The p4Δ*cps2N* plasmid was then introduced into the competent SS2Δsynth strain. Electroporation and SS2Δsynth/ΔsiaT (SS2Δ*neu2C*/Δ*cps2N*) double mutant construction were carried out under the same aforementioned conditions (Roy et al., 2016).

Finally, in order to reintroduce a functional *neuC* gene into the double mutant SS2Δsynth/ΔsiaT *(*Δ*neu2C*/Δ*cps2N*), the intact *neuC* gene with corresponding upstream/downstream regions of *neuC* was amplified by PCR from P1/7 strain and cloned into the pSET4s vector as described above, giving rise to p4*neuC*. The p4*neuC* plasmid was then introduced into the competent double mutant SS2Δsynth/ΔsiaT *(*Δ*neu2C*/Δ*cps2N*) to obtain the indirect deletion mutant SS2ΔsiaT (Δ*cps2N*). Electroporation and mutant construction were carried out as already described (Roy et al., 2016). Deletion/insertion of targeted genes was confirmed by PCR and sequence analysis. Moreover, giving the fact that mutations in the *cps* locus (mostly in *cps2E* and *cps2F*) can occur during such deletion (Lakkitjaroen et al., 2014), we also sequenced *cps2E* and *cps2F* genes in SS2ΔsiaT mutant and confirmed that no mutation was present.

### Indirect substitution of the sialyltransferase gene (*cpsN*) in *s. suis* serotypes 2 and 14

To obtain sialyltransferase substitution mutants in *S. suis* serotypes 2 and 14, we used the same approach as for the indirect deletion of *cps2N*. Precise in-frame deletion in *neu2C* (serotype 2) and *neu14C* (serotype 14) genes (UDP-*N*-acetylglucosamine 2-epimerase; sialic acid synthesis) was achieved using the p4Δ*neuC* mutation vector as described above. The same vector (p4Δ*neuC*) was used for both serotypes 2 and 14, as corresponding sequences were 100% identical. Again, this step was required in order to inhibit CPS production and thus by-pass the lethality induced by mutation of the sialyltransferase gene in *S. suis* (Lakkitjaroen et al., 2014).

Gene substitution plasmids p4sia2,3_2 and p4sia2,3_14 were constructed by overlapping PCR, merging the intact *cps3K* gene (coding for the GBS α-2,3-sialyltransferase) with upstream and downstream coding regions of *cps2N* and *cps14N* genes (coding for the *S. suis* α-2,6-sialyltransferases). Overlapping PCR products generated by PCR were then cloned into the thermosensitive pSET4s vector. Resulting substitution plasmids p4sia2,3_2 and p4sia2,3_14 were introduced into competent *S. suis* SS2Δsynth (serotype 2) and SS14Δsynth (serotype 14) mutants under the same aforementioned electroporation conditions. Substitution mutants SS2Δ*neu2C*/*cps3K* (SS2Δsynth/sia2,3) and SS14Δ*neu14C*/*cps3K* (SS14Δsynth/sia2,3) were obtained as described for other *S. suis* mutants (Roy et al., 2016).

Finally, in order to reintroduce a functional *neuC* gene into SS2Δ*neu2C*/*cps3K* and SS14Δ*neu14C*/*cps3K* mutants, the p4*neuC* plasmid was introduced into competent SS2Δ*neu2C*/*cps3K* and SS14Δ*neu14C*/*cps3K* mutants to obtain indirect substitution mutants SS2sia2,3 and SS14sia2,3. The same vector (p4*neuC*) was used for both serotypes 2 and 14, as corresponding sequences were 100% identical. Electroporation and mutant construction were carried out as described above. Gene substitution was confirmed by PCR and sequence analysis. As for SS2ΔsiaT mutant, we also sequenced *cpsE* and *cpsF* genes in SS2Δ*neu2C*/*cps3K* and SS14Δ*neu14C*/*cps3K* mutants and confirmed absence of mutations.

### Construction of the complemented Δ*cps2N* mutant (SS2compΔsiaT)

The intact *cps2N* gene was amplified from genomic DNA of *S. suis* serotype 2 wild-type strain with primers containing specific restriction sites (Supplementary Table S1). PCR products and pMX1 vectors were then digested with the appropriate restriction enzyme before ligation. Final constructions were cloned into *E. coli* MC1061. The plasmid pMX1 is a derivative of the *S. suis*–*E. coli* shuttle cloning vector pSET2 and possesses the *S. suis* malQ promoter for transgene expression in *S. suis* (Takamatsu et al., 2001a). Complementation of the SS2ΔsiaT (Δ*cps2N*) mutant was achieved by transformation with the pMX1*cps2N* complementation vector by electroporation under the same aforementioned conditions. Presence of the plasmid within the complemented mutant was confirmed by PCR. Plasmid stability was evaluated by measuring bacterial growth rates under selective pressure (Sp; 100 μg/ml). As for SS2ΔsiaT mutant, we sequenced *cps2E* and *cps2F* genes to confirm that no mutations occurred during transformation procedures.

### Deletion mutants in GBS type V

Precise in-frame deletion in *cps5K* (GBSVΔsiaT) and *neu5B* (GBSVΔsynth) genes was achieved using splicing-by-overlap-extension PCR (Warrens et al., 1997). Overlapping PCR products generated by PCR were cloned into the plasmid pCR2.1 (Invitrogen), extracted using restriction-enzyme digestion, and cloned into the thermosensitive *E. coli-S. suis* shuttle vector pSET4s, giving rise to the p4Δ*cps5K* and p4Δ*neu5B* mutation vectors. Final constructions of the pSET4s vector (p4Δ*cps5K* and p4Δ*neu5B*) were electroporated into GBS type V competent cells. Electroporation and mutant construction were carried out as described previously for *S. suis* (Roy et al., 2016). Deletions of the *cps5K* and *neu5B* genes in GBSVΔsiaT and GBSVΔsynth, respectively, were confirmed by PCR and sequence analysis.

### Exogenous sialyltransferase exchange in GBS types III and V

In contrast to *S. suis*, in order to substitute GBS type III and V sialyltransferases, direct gene replacement by double-crossover homologous recombination system was used. The upstream region and downstream region of GBS sialyltransferase *cps3K* or *cps5K* genes (accession numbers: AAD53072 and NP_688172, respectively) were amplified by PCR, conserving the intact stop codon of the upstream coding gene and the intact start codon of the downstream coding gene. In addition, the complete intact *cps2N* sialyltransferase gene (accession number: CAR45180) of *S. suis* serotype 2 strain P1/7 was amplified by PCR. The PCR products were then merged together by overlapping PCRs. The cloning step in pCR2.1 and pSET4s was done as described above, giving rise to substitution vectors p4sia2,6_III and p4sia2,6_V. Final constructions (p4sia2,6_III and p4sia2,6_V) were introduced into competent GBS under the same electroporation conditions than for *S. suis*. Substitution mutants GBSIIIsia2,6 and GBSVsia2,6 were obtained as described for GBS deletion mutants. Gene substitution was confirmed by PCR and sequence analysis.

### Hydrophobicity test

In order to have a qualitative first impression of CPS expression by different mutant strains, *S. suis* and GBS mutants were tested (triplicate independent assays) for cell surface hydrophobicity by measuring their absorption to *n*-hexadecane according to the procedure previously described (Bonifait et al., 2010). Reference strains of *S. suis* serotype 2 (P1/7), *S. suis* serotype 14 (DAN13730), GBS serotype III (COH1), and GBS serotype V (CJB111) were used as positive controls. Non-encapsulated mutants SS2Δ*cps*, SS14Δ*cps*, GBSIIIΔ*cps*, and GBSVΔ*cps* were used as reference for a non-encapsulated phenotype. Since the hydrophobicity test has limited sensitivity, other methods were also used to confirm CPS expression (see sections Transmission Electron Microscopy (TEM), CPS Purification From GBS Mutants, Nuclear Magnetic Resonance (NMR) Spectroscopy, and Weight-Average Molecular Mass Characterization of Purified CPSs).

### Whole-bacterial cell enzyme-linked lectin assay (ELLA)

In order to investigate the specific linkage of sialic acid in the sialyltransferase substitution mutants, a whole-bacterial cell ELLA was carried out with the biotinylated *Sambucus nigra* agglutinin (SNA-I, Vector Labs Canada, Burlington, ON, Canada) and the biotinylated *Maackia amurensis* leukoagglutinin (MAL-I, Vector Labs) which specifically recognize sialic acid as Neu5Acα-2,6-Gal*p*/Gal*p*NAc or as Neu5Acα-2,3-Galβ-1,4-GlcNAc, respectively (Shibuya et al., 1987; Geisler and Jarvis, 2011). The test was based on a previously described technique and adapted for whole bacteria (Gornik and Lauc, 2007). A 10-ml overnight culture in THB inoculated with the appropriate strains was harvested by centrifugation, washed, and resuspended in 10 ml of PBS. The suspension was then diluted 10X in PBS (10^7^ CFU/ml), and 100 μl was distributed into wells of an ELISA plate (Nunc-Immuno Polysorp, Canadawide Scientific, Toronto, ON, Canada). Wells were then dried overnight, fixed with 50 μl of 100% high-quality methanol, and dried for 20 min. After coating, the wells were washed and blocked by the addition of Carbo-Free solution 1X (Vector Labs). After washing, the wells were incubated 1 h with biotinylated SNA-I or biotinylated MAL-I followed by horseradish peroxidase-labeled Avidin D (Vector Labs), and 3,3′,5,5′-tetramethylbenzidine was finally added for detection. The enzyme reaction was stopped with the addition of 1 N H_2_SO_4_, and the absorbance was read at 450 nm with an ELISA plate reader.

### Transmission electron microscopy (TEM)

TEM was carried out to confirm CPS expression at the bacterial surface of different mutant strains as previously described (Roy et al., 2015). Briefly, bacteria were grown to mid-logarithmic phase and resuspended in 0.1 M cacodylate buffer pH 7.3 containing 2.5% (v/v) glutaraldehyde and 0.05% (w/v) ruthenium red. Fixation was performed for 2 h at room temperature. Ferritin (Electron Microscopy Sciences, Hatfield, PA) was then added to a final concentration of 1 mg/ml and incubated for 30 min at room temperature. Afterwards, cells were immobilized in 4% (w/v) agar in 0.1 M cacodylate buffer pH 7.3 and post-fixed with 2% (v/v) osmium tetroxide in water overnight at 4°C. Samples were washed with water every 20 min for 2 h to remove osmium tetroxide and dehydrated in an increasing graded series of acetone. Specimens were then washed twice in propylene oxide and embedded in Spurr low-viscosity resin (Electron Microscopy Sciences). Thin sections were post-stained with uranyl acetate and lead citrate and examined with a transmission electron microscope at 80 kV (model JEM 1230, Jeol, Tokyo, Japan).

### CPS purification from GBS mutants

CPSs from the GBS type III mutant GBSIIIsia2,6 and GBS type V mutants GBSVsia2,6, GBSVΔsynth, and GBSVΔsiaT were purified as previously described (Calzas et al., 2013). Briefly, 8 l of THB (Oxoid, Thermo Fisher Scientific, ON, Canada) was inoculated with an overnight culture of the appropriate strain (1:40 dilution) and incubated until OD_540_ reached 0.8. The bacterial cells were harvested by centrifugation at 10,000 *g* for 40 min, washed in PBS pH 7.3, and treated with 1 N NaOH at 37°C overnight. After neutralization and dialysis, proteins were digested with 1 mg/ml pronase (Sigma) at 37°C overnight. After subsequent dialysis, the CPSs were treated with 0.8 M acetic anhydride (Sigma) in 5 N NaOH for re-*N*-acetylation of polysaccharides. The CPSs were finally purified by gel filtration on Sephacryl S-300 (GE Healthcare, Little Chalfont, UK), using 50 mM NH_4_HCO_3_ as the eluent. Control native CPSs were also purified from respective wild-type strains.

### Nuclear magnetic resonance (NMR) spectroscopy

Purified CPSs from GBSVΔsynth, GBSVΔsiaT, GBSVsia2,6, and GBSIIIsia2,6 mutants were exchanged in phosphate buffer pD 8.0 in D_2_O (99.9 atom% D), freeze dried, and dissolved in D_2_O (99.96 atom% D) to a final concentration of 33 mM. The other polysaccharides were exchanged in D_2_O (99.9 atom% D), freeze dried, and dissolved in D_2_O (99.96 atom% D). NMR spectra were acquired on polysaccharide samples at concentrations of 0.1–1.1%. ^1^H chemical shifts δ in ppm were referenced to internal deuterated 2,2-dimethyl-2-silapentane-5-sulfonate (DSS-*d*_6_) at δ 0 as recommended by Wishart et al. (1995). A Chemagnetics (Fort Collins, CO) CMX Infinity 300 spectrometer was used for 7.05-T experiments with a 5-mm dual ^13^C/^1^H Nalorac probe (Martinez, CA) at 60°C. The one-dimensional (1D) ^1^H experiment was performed with the original pulse program of the Spinsight software. The 16 K complex data points were acquired and processed by exponential multiplication with a line broadening factor equal to the digital resolution, complex Fourier transform, phase correction, and fifth-order polynomial baseline correction. Alternatively, spectra were acquired at 11.75 T on a Bruker Avance 500 spectrometer equipped with a 5-mm triple resonance TBI probe with ^1^H, ^13^C, and ^109^Ag–^31^P channels at 60–80°C or at 16.45 T on a Bruker Avance 700 spectrometer with a 5-mm cryoprobe with ^1^H and ^13^C channels at 65°C using standard Bruker pulse sequences at the Centre régional de résonance magnétique nucléaire (Department of Chemistry, University of Montreal). Conventional 1D ^1^H spectra were acquired with 90 or 30° pulses with or without solvent presaturation. The *z*-restored spin-echo was used to acquire 1D ^1^H-decoupled ^13^C spectrum of straight baseline. The 1D distortionless enhancement by polarization transfer (DEPT) spectrum with adiabatic pulses was recorded with a reading pulse of 135° and the free-precession period optimized for 145-Hz one-bond coupling constant. The gradient-enhanced two-dimensional (ge-2D) correlation spectroscopy (COSY) spectrum was acquired in magnitude mode. The phase-sensitive 2D total correlation spectroscopy (TOCSY) spectrum with Malcom Levitt's sequence (MLEV) was acquired with or without presaturation and an effective spin lock time of 80 ms. The phase-sensitive 2D rotating-frame nuclear Overhauser spectroscopy (ROESY) spectrum with presaturation or using purging pulses was acquired with a mixing time of 300 ms. The phase-sensitive ge-2D heteronuclear single-quantum coherence (HSQC) experiment using echo–antiecho and adiabatic pulses for inversion and refocusing and Bloch-Siegert effects was optimized for 145–155 Hz. The phase-sensitive ge-2D HSQC–TOCSY experiment with MLEV using echo–antiecho was performed with a delay optimized for a 140–145-Hz coupling constant and a mixing time of 80 ms. The phase-sensitive ge-2D heteronuclear multiple-bond correlation (HMBC) experiment using a three-fold low-pass *J*-filter was run without ^13^C decoupling with one-bond and long-range delays optimized for 145 and 1–8 Hz, respectively. Bruker spectra were processed off-line with SpinWorks (Copyright, Kirk Marat, University of Manitoba [http://home.cc.umanitoba.ca/~wolowiec/spinworks/]). For 1D spectra, 29–64 K complex data points were acquired and processed by exponential multiplication with a line broadening factor equal to the digital resolution, zero filling, complex Fourier transform, phase correction, and fifth-order polynomial baseline correction. Zhu-Bax forward–backward linear prediction with 16 coefficients was systematically applied to 2D processing in the *f*_1_ dimension (Zhu, 1992).

### Weight-average molecular mass characterization of purified CPSs

The weight-average molecular mass (*M*_w_) of each CPS was determined by size-exclusion chromatography coupled with multi-angle light scattering (SEC-MALS) as described by Calzas et al. (2013). The chromatographic separation was performed with two 8 × 300 mm Shodex OHpak gel filtration columns connected in series (SB-806 and SB-804), preceded by an SB-807G guard column (Showa Denko, Tokyo, Japan). Elution was done with a Waters 510 pump (Waters, Milford, MA), using a 0.1 M NaNO_3_ mobile phase filtered through a 0.02-μm membrane (Whatman, Maidstone, UK), at a flow rate of 0.5 ml/min. Samples were dissolved in the SEC eluent at concentrations of 0.7–1.0 mg/ml and then injected with a 100 or 200-μl sample loop. Molecular masses were determined with a Dawn EOS MALS detector (Wyatt, Santa Barbara, CA). A model RI 410 differential refractometer (Waters) was used as a concentration detector. A refractive index increment (*dn*/*dc*) of 0.137 ml/g was calculated for 690 nm, using data for xanthan at 436 and 546 nm, and the second virial coefficient (*A*_2_) was taken as zero. Calculations were performed with the ASTRA software, version 6.0.0.108 (Wyatt).

### Statistical analysis

All data are expressed as mean ± SEM and were analyzed for significance using the Student's *t*-test. Normality was previously verified in order to select the appropriate test. A *P*-value < 0.05 was used as a threshold for significance.

## Results

### Transcription of the *S. suis cps* locus occurs in a single polycistronic transcript

**Figure 3:**
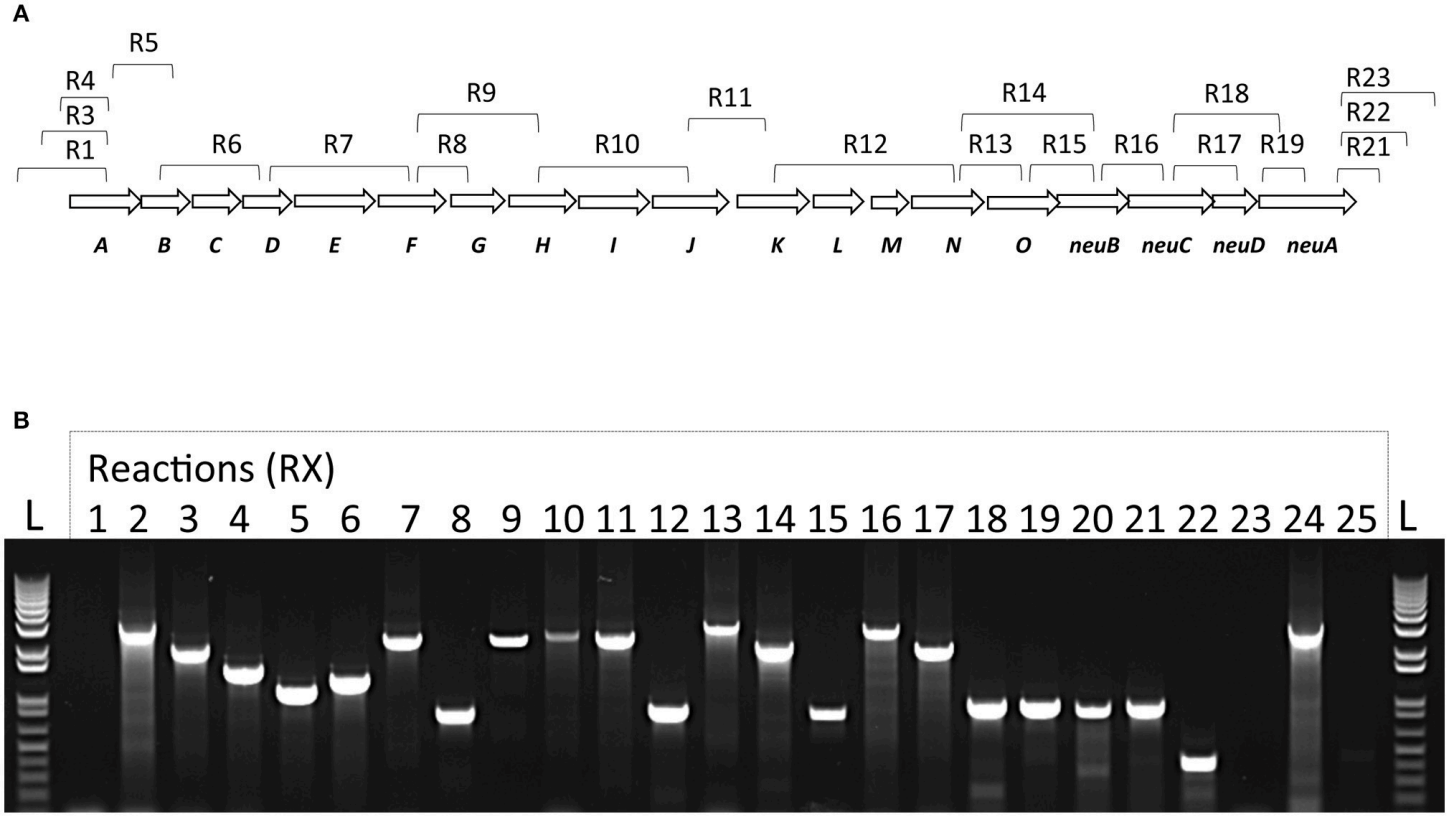
Transcriptional analysis of the *S. suis cps* operon. **(A)** Schematic representation of *S. suis* capsular polysaccharide synthesis coding region with corresponding RT-PCR reactions and **(B)** agarose gel electrophoresis of RT-PCR reaction products visualized under UV light. Total bacterial RNA was isolated and converted to cDNA. RNA samples without reverse-transcription step were used as template to verify absence of genomic DNA in lane R25. Genomic DNA with the same primers was used as positive control (lane R24). All RT-PCR products migrated accordingly to their expected sizes. DNA size standards (“L”) are depicted at the left- and right-end sides.

To confirm that the *cps* locus of *S. suis* is encoding a single polycistronic transcript and to confirm that the sialyltransferase is under the same promoter than other CPS synthesis genes, RNA obtained from an overnight culture of *S. suis* was analyzed by RT-PCR. Using appropriate primers from adjacent genes (Supplementary Table S1), we showed that all genes within the *cps* coding locus are transcripted as a single polycistronic transcript (Figure 3). Indeed, RT-PCR products for all primers within the *cps* locus were obtained. Negative RT-PCR amplifications were obtained with reactions R1 and R23, delimiting the mRNA transcript. Genomic DNA was used to confirm primer efficiency for R1 and R23 as shown with R2 and R24, respectively. No amplified products were present in negative-control reaction (R25) without RT. These results confirmed that the *cps* locus of *S. suis* is encoding a single polycistronic transcript under the regulation of the same promoter (Figure 3).

### Deletion of the sialyltransferase in *S. suis* results in a non-encapsulated phenotype

**Figure 4:**
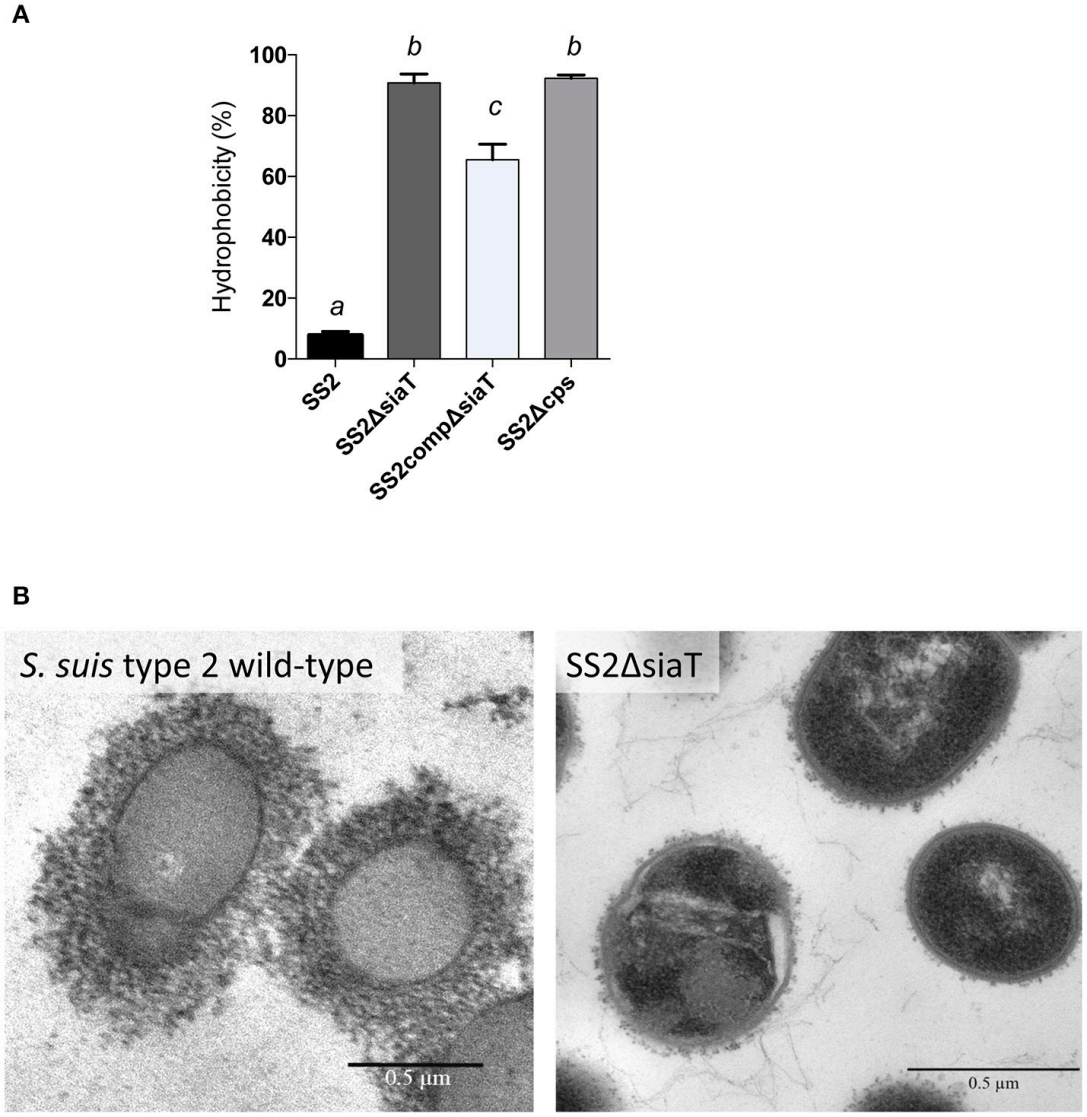
Capsular polysaccharide (CPS) expression levels of *S. suis* serotype 2 and derived isogenic mutants. **(A)** Hydrophobicity (%) of the wild-type *S. suis* serotype 2 strain (SS2), the sialyltransferase SS2ΔsiaT (Δ*cps2N*) mutant, the complemented SS2compΔsiaT mutant, and of the non-encapsulated strain (SS2Δ*cps*) used as control. Data are expressed as mean ± SEM of at least three independent experiments. Student's *t*-test analyses reported significant differences between “*a”* and “*b”*, between “*a”* and “*c”*, and between “*b”* and “*c”* (*P* < 0.05). **(B)** Transmission electron micrographs showing CPS expression by *S. suis* serotype 2 wild-type strain and its sialyltransferase SS2ΔsiaT (Δ*cps2N*) mutant. Bars = 0.5 μm.

It has been previously reported that deletion of the *neuC* gene (UDP-*N*-acetylglucosamine 2-epimerase; sialic acid synthesis) in *S. suis* serotypes 2 and 14 results in a non-encapsulated phenotype (Table 1). However, the phenotypic outcome of a deletion of the sialyltransferase (*cpsN*) gene has never been addressed. Given the fact that mutations blocking side-chain assembly (*cpsJ*), polymerization (*cpsL*), sialylation (*cpsN*), or exportation (*cpsO*) are lethal for *S. suis* (Lakkitjaroen et al., 2014), we developed a three-step mutagenesis approach in order to by-pass the lethality of sialyltransferase (*cpsN*) mutation. It has been shown that mutation in the sialyltransferase can occur naturally in *S. suis* in presence of a suppressive mutation in other CPS synthesis genes that results in CPS inhibition (Lakkitjaroen et al., 2014). We thus took advantage of this particularity in order to knockout the sialyltransferase gene in *S. suis* serotype 2 (mutant SS2ΔsiaT; see experimental procedures). As depicted in Figure 4A, the hydrophobicity of the SS2ΔsiaT mutant was very similar to that of the non-encapsulated control strain (SS2Δ*cps*), whereas the well-encapsulated wild-type serotype 2 strain P1/7 showed very low hydrophobicity. As expected, the complemented SS2ΔsiaT mutant (SS2compΔsiaT) showed partially reduced hydrophobicity levels when compared to the SS2ΔsiaT mutant (*P* = 0.0027). Despite the pMX1 vector was successfully used in several complementation studies, complementation in *S. suis* with pMX1 results in an intermediate state when compared to deficient mutant and wild-type strain (Lecours et al., 2012; Roy et al., 2015; Ferrando et al., 2017). Absence of CPS expression by the SS2ΔsiaT mutant was also confirmed by TEM, as shown in Figure 4B, where a total loss of CPS expression was observed in the mutant strain compared to an apparent thick CPS surrounding the wild-type strain.

### Substitution of the α-2,6-sialyltransferase by the GBS α-2,3-sialyltransferase in *S. suis* serotypes 2 and 14 also results in non-encapsulated phenotypes

**Figure 5:**
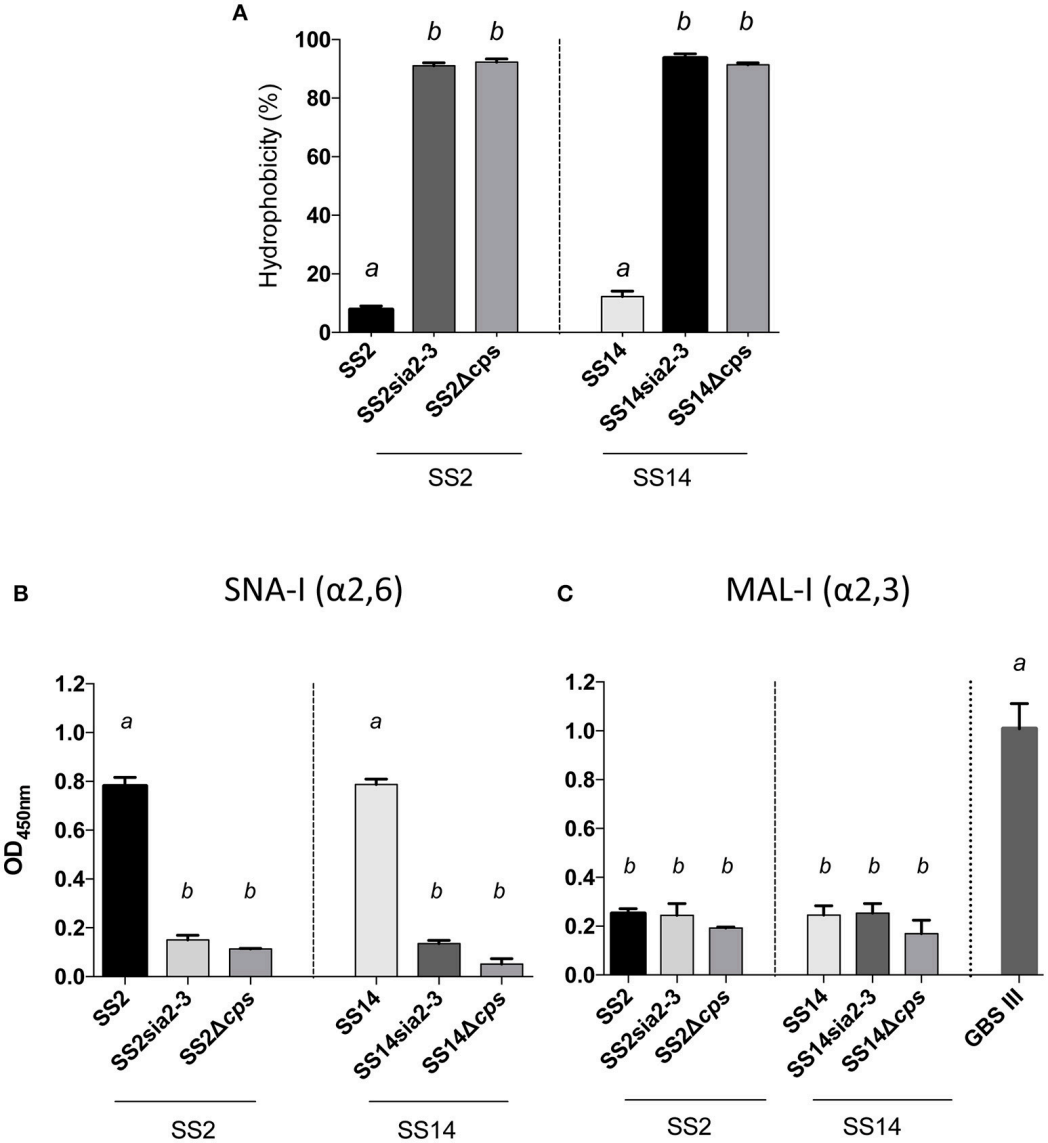
Capsular polysaccharide (CPS) expression levels and sialic acid linkage in *S. suis* serotype 2 and 14 mutants carrying exogenous α-2,3-sialyltransferase. **(A)** Hydrophobicity (%) of the wild-type *S. suis* serotype 2 (SS2) and 14 (SS14) strains, the SS2sia2,3 (Δ*cps2N*/*cpsK*) and SS14sia2,3 (Δ*cps14N*/*cpsK*) mutants carrying the GBS α-2,3-sialyltransferase (*cpsK*). The non-encapsulated mutants SS2Δ*cps* and SS14Δ*cps* were used as control strains. **(B,C)** Whole-bacterial cell enzyme-linked lectin assay (ELLA) was performed to detect α-2,3 or α-2,6 capsular sialic acid linkage in SS2sia2,3 and SS14sia2,3 mutant strains. Whole bacteria were incubated with *Sambucus nigra* agglutinin (SNA-I) specific for Neu5Ac α-2,6 linkages, or *Maackia amurensis* leukoagglutinin (MAL-I) specific for Neu5Ac α-2,3 linkages. The non-encapsulated mutants SS2Δ*cps* and SS14Δ*cps* were used as negative controls. SS2 was used as positive control for SNA-I and wild-type GBS type III as positive control for MAL-I. Data in **(A–C)** are expressed as mean ± SEM of at least three independent experiments. Student's *t*-test analyses reported significant differences between “*a”* and “*b”* (*P* < 0.05).

**Figure 6:**
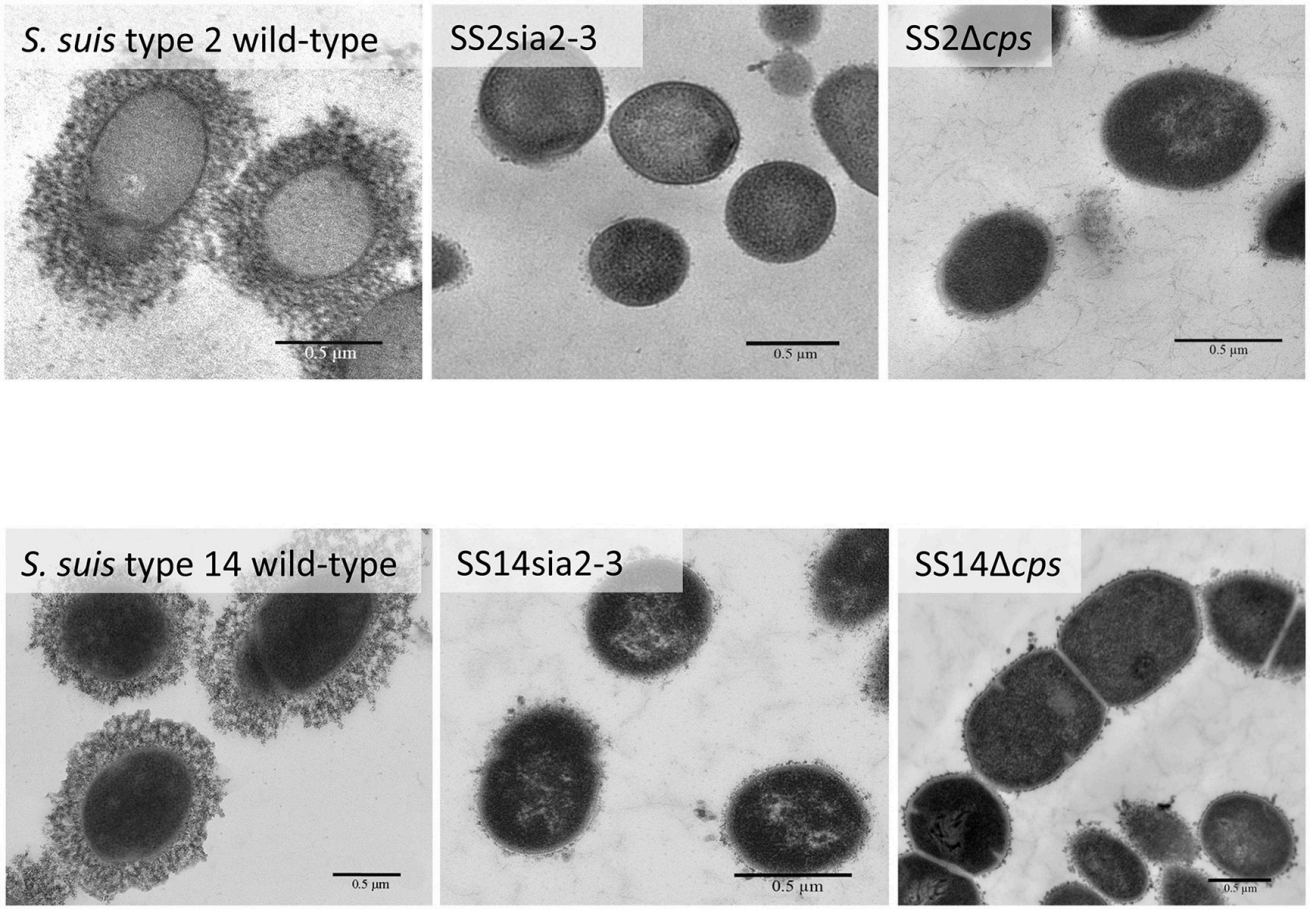
Transmission electron micrographs showing capsular polysaccharide (CPS) expression by *S. suis* serotype 2 and 14 isogenic mutants. The CPS was labeled with polycationic ferritin. *S. suis* serotype 2 wild-type strain and *S. suis* serotype 14 wild-type strain were surrounded by a thick capsule, whereas the SS2sia2,3 (Δ*cps2N*/*cpsK*) and the SS14sia2,3 (Δ*cps14N*/*cpsK*) were non-encapsulated. Non-encapsulated mutant strains SS2Δ*cps* and SS14Δ*cps* were included as negative controls. Bars = 0.5 μm.

In order to better dissect the importance and specificity of the sialyltransferase for CPS expression at the bacterial surface, we substituted the *S. suis* α-2,6-sialyltransferase by the GBS α-2,3-sialyltransferase using the same mutagenesis approach. The *S. suis* serotype 2 mutant SS2sia2,3 and the *S. suis* serotype 14 mutant SS14sia2,3 both showed very high hydrophobicity, which was similar to that of respective non-encapsulated mutant strains (Figure 5A). In addition, we investigated the presence of sialic acid and its linkage (if present) by an ELLA using α-2,6- or α-2,3-specific lectins. As shown in Figures 5B,C, *S. suis* serotype 2 and 14 mutants carrying the GBS α-2,3-sialyltransferase (SS2sia2,3 and SS14sia2,3) presented negative reactions with both SNA-I lectin (α-2,6) and MAL-I lectin (α-2,3), suggesting total absence of sialic acid at the bacterial surface. Consistent with hydrophobicity test results, TEM analysis confirmed the non-encapsulated phenotypes of mutants SS2sia2,3 and SS14sia2,3 (Figure 6).

### Deletion of sialyltransferase or sialic acid synthase genes in GBS type V results in asialo encapsulated phenotype

**Figure 7:**
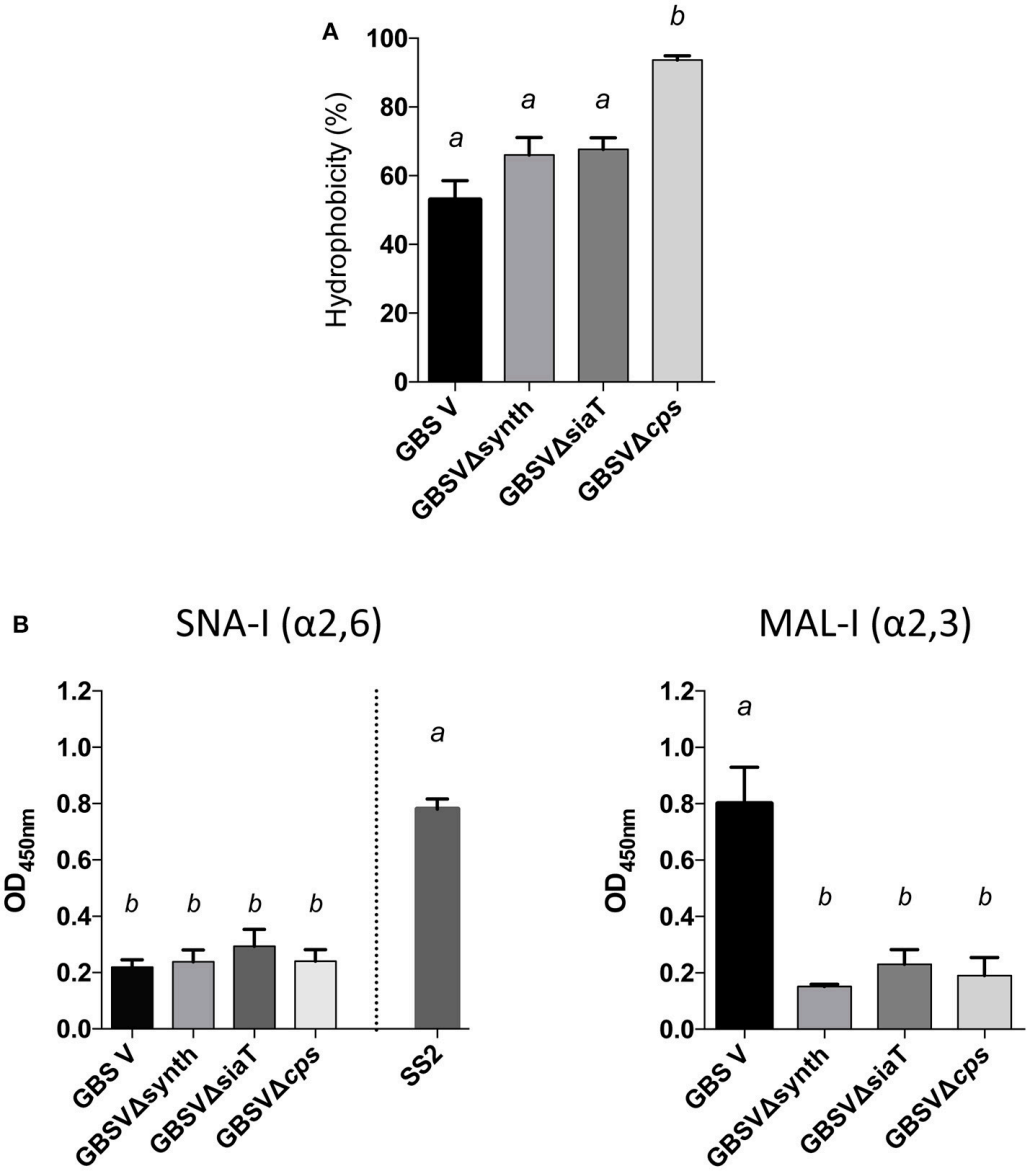
Capsular polysaccharide (CPS) expression levels and recognition of specific CPS sialic acid linkage in GBS type V isogenic mutants. **(A)** Hydrophobicity (%) of the wild-type GBS serotype V strain (GBS V), and the sialic acid synthesis GBSVΔsynth (Δ*neu5B*) and sialyltransferase GBSVΔsiaT (Δ*cps5K*) deficient mutants. The non-encapsulated strain (GBSVΔ*cps*) was used as control. **(B)** Whole-bacterial cell enzyme-linked lectin assay (ELLA) was performed to detect α-2,3 or α-2,6 capsular sialic acid linkage in these mutant strains. Whole bacteria were incubated with *Sambucus nigra* agglutinin (SNA-I) specific for Neu5Ac α-2,6 linkages, or *Maackia amurensis* leukoagglutinin (MAL-I) specific for Neu5Ac α-2,3 linkages. The non-encapsulated mutant GBSVΔ*cps* was used as negative control. *S. suis* serotype 2 (SS2) was used as positive control for SNA-I and wild-type GBS type V as positive control for MAL-I. Data in **(A,B)** are expressed as mean ± SEM of at least three independent experiments. Student's *t*-test analyses reported significant differences between “*a”* and “*b”* (*P* < 0.05).

**Figure 8:**
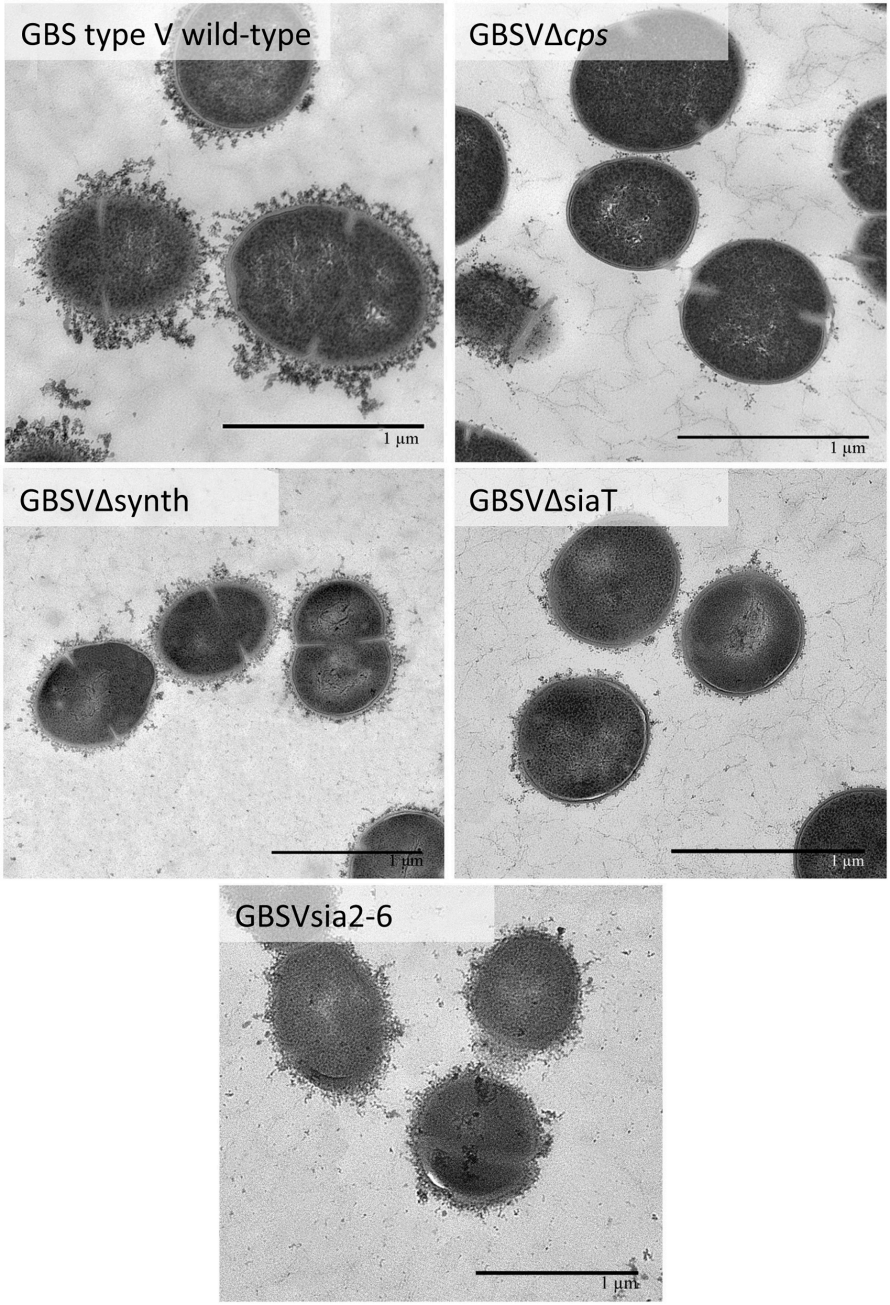
Transmission electron micrographs showing capsular polysaccharide (CPS) expression by GBS type V isogenic mutants. The CPS was labeled with polycationic ferritin. GBS type V wild-type strain was surrounded by a consistent CPS layer, whereas the GBSVΔsiaT (Δ*cps5K*), the GBSVΔsynth (Δ*neu5B*), and the GBSVsia2,6 (Δ*cps5K*/*cps2N*) mutants all showed intermediate state of encapsulation. The non-encapsulated mutant strain GBSVΔ*cps* is depicted as negative control. Bars = 1 μm.

In order to determine if the non-encapsulated phenotype resulting from deletion of the sialic acid synthesis gene or deletion/substitution of the sialyltransferase gene is specific to *S. suis*, we investigated for the first time the role of sialic acid in CPS expression by GBS type V. In contrast to *S. suis*, deletion of the sialyltransferase gene (*cps5K*) or the sialic acid synthesis gene (*neu5B*) of GBS type V had no inhibitory effect on CPS expression at the bacterial surface. Indeed, as shown in Figure 7A, the sialyltransferase mutant GBSVΔsiaT (Δ*cps5K*) and the sialic acid synthase mutant GBSVΔsynth (Δ*neu5B*) possessed moderate hydrophobicity, which was indeed similar to that of the encapsulated wild-type strain and significantly lower to that obtained with the non-encapsulated type V mutant used as control (*P* = 0.0063 for GBSVΔsynth and *P* = 0.0019 for GBSVΔsiaT). These results suggest similar CPS expression between wild-type strain and both mutants (GBSVΔsiaT and GBSVΔsynth). The ELLA showed negative reactions with both SNA-I and MAL-I lectins (Figure 7B), suggesting total absence of sialic acid in the CPS produced by GBSVΔsiaT and GBSVΔsynth mutants. TEM analyses were used to confirm the presence of CPS in these two mutant strains. As depicted in Figure 8, mutants GBSVΔsiaT and GBSVΔsynth showed intermediate levels of encapsulation when compared to the serotype V wild-type strain and the non-encapsulated mutant GBSVΔ*cps*. Using the same CPS purification protocol for all strains, the CPS yield recovered from mutants GBSVΔsiaT and GBSVΔsynth was reduced when compared to wild-type GBS type V (Table 2), confirming intermediate levels of encapsulation in the two mutant strains. Analyses of purified CPSs by SEC-MALS also showed reduced weight-average molecular mass (*M*_w_) for the two mutant strains derived CPSs when compared to the wild-type strain (Table 2).

**Table 2:** Group B *Streptococcus* (GBS) capsular polysaccharide (CPS) yields and weight-average molecular mass.

**GBS strain**	**CPS type**	**Average yield (mg)**	***M*_w_ (kg/mol)^a^**
GBS III wild type	III	50.4^b^	108.0^b^
GBSIIIsia2,6	III	12.0	44.7
GBS V wild type	V	50.5^b^	128.0^b^
GBSVsia2,6	V	15.0	69.4
GBSΔsiaT	V	7.0	58.1
GBSΔsynth	V	16.0	62.4

a *M_w_, weight-average molecular mass, determined by size-exclusion chromatography coupled with multi-angle light scattering (SEC-MALS)*.

b *Published in Calzas et al. (2013)*.

**Figure 9:**
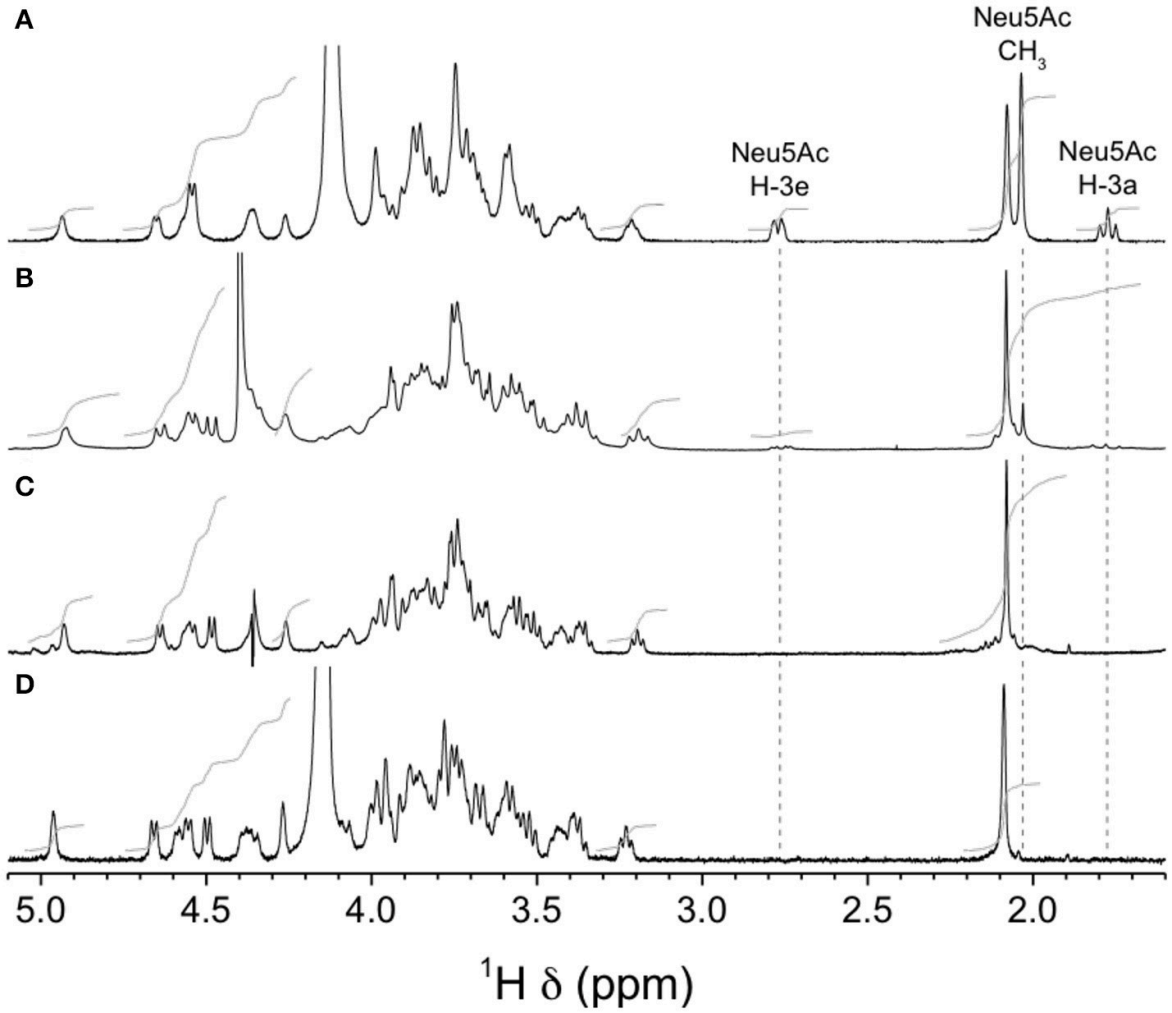
H NMR spectra of native GBS type V and sialic acid-deficient mutant capsular polysaccharides (CPSs). **(A)** Wild-type, native CPS, D_2_O, 500 MHz, 30° pulse, 80°C. **(B)** GBSVΔsynth (Δ*neu5B*) mutant, native CPS, D_2_O, 300 MHz, 90° pulse, 60°C. **(C)** GBSVΔsiaT (Δ*cps5K*) mutant, native CPS, 33 mM phosphate pD 8.0 in D_2_O, 500 MHz, 90° pulse with presaturation, 67°C. **(D)** Wild-type, chemically desialylated polysaccharide, D_2_O, 500 MHz, 30° pulse, 80°C.

Finally, we analyzed purified CPSs by NMR to confirm the absence of sialic acid in mutants GBSVΔsiaT and GBSVΔsynth. Integration of sialic acid reporter resonance signals (i.e., H-3e at δ 2.76, H-3a at δ 1.77, and *N*-acetyl CH_3_ at δ 2.03) in the ^1^H spectrum of the GBSVΔsynth (Δ*neu5B*) mutant CPS (Figure 9B) represented *ca*. 0.2 equivalent compared to that of the wild-type GBS type V CPS (Figure 9A). These signals were totally absent from the spectrum of the GBSVΔsiaT (Δ*cps5K*) mutant CPS (Figure 9C), which in fact was essentially identical to that of the chemically desialylated GBS type V polysaccharide (Figure 9D; Calzas et al., 2013).

### Substitution of the GBS α-2,3-sialyltransferase by the *S. suis* α-2,6-sialyltransferase in GBS results in successfully modified sialic acid linkage

**Figure 10:**
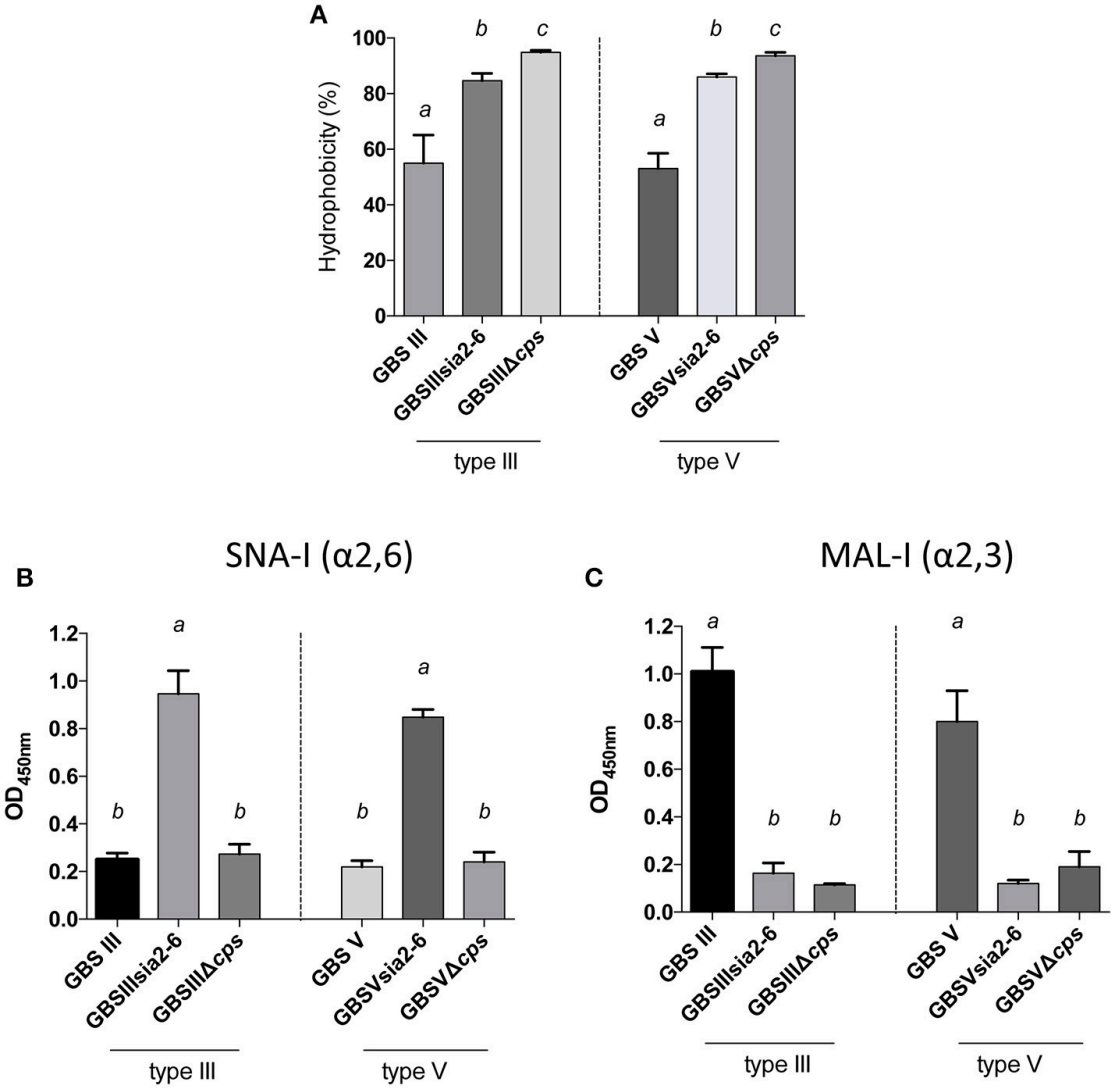
Capsular polysaccharide (CPS) expression levels and sialic acid linkage in GBS type III and V mutants carrying exogenous α-2,6-sialyltransferase. **(A)** Hydrophobicity (%) of GBS type III and V wild-type strains and the mutants GBSIIIsia2,6 (Δ*cps3K*/*cps2N*) and GBSVsia2,6 (Δ*cps5K*/*cps2N*) carrying the *S. suis* α-2,6-sialyltransferase. The non-encapsulated mutant strains (GBSIIIΔ*cps* and GBSVΔ*cps*) were used as controls. **(B,C)** Whole-bacterial cell enzyme-linked lectin assay (ELLA) was performed to detect α-2,3 or α-2,6 capsular sialic acid linkage in these mutant strains. Whole bacteria were incubated with *Sambucus nigra* agglutinin (SNA-I) specific for Neu5Ac α-2,6 linkages, or *Maackia amurensis* leukoagglutinin (MAL-I) specific for Neu5Ac α-2,3 linkages. The non-encapsulated mutants were used as negative controls. Data in **(A–C)** are expressed as mean ± SEM of at least three independent experiments. Student's *t*-test analyses reported significant differences between “*a”* and “*b”*, between “*a”* and “*c”*, and between “*b”* and “*c”* (*P* < 0.05).

In order to study the specific role of sialic acid linkage in CPS expression by GBS, we substituted GBS type III and V sialyltransferase *cpsK* genes by the *S. suis* sialyltransferase gene *cpsN*. We first evaluated CPS expression by the hydrophobicity test. As shown in Figure 10A, mutants with modified sialyltransferase GBSIIIsia2,6 and GBSVsia2,6 presented high hydrophobicity when compared to the wild-type strain, but significantly lower than the non-encapsulated mutant (*P* = 0.0473 for GBSIIIsia2,6 and *P* = 0.0046 for GBSVsia2,6), suggesting the presence of reduced amount of capsule at the bacterial surface. The ELLA (Figures 10B,C) was used to verify the sialic acid linkage obtained after sialyltransferase gene substitution. Mutants GBSIIIsia2,6 and GBSVsia2,6 showed positive reactions with SNA-I (α-2,6) lectin when compared to GBS wild-type strains (*P* = 0.0016 for GBSIIIsia2,6 and *P* = 0.0019 for GBSVsia2,6; Figure 10B). In addition, negative reactions were observed for GBSIIIsia2,6 and GBSVsia2,6 with MAL-I (α-2,3) lectin, suggesting the expression of a CPS with modified sialic acid linkage (Figure 10C).

**Figure 11:**
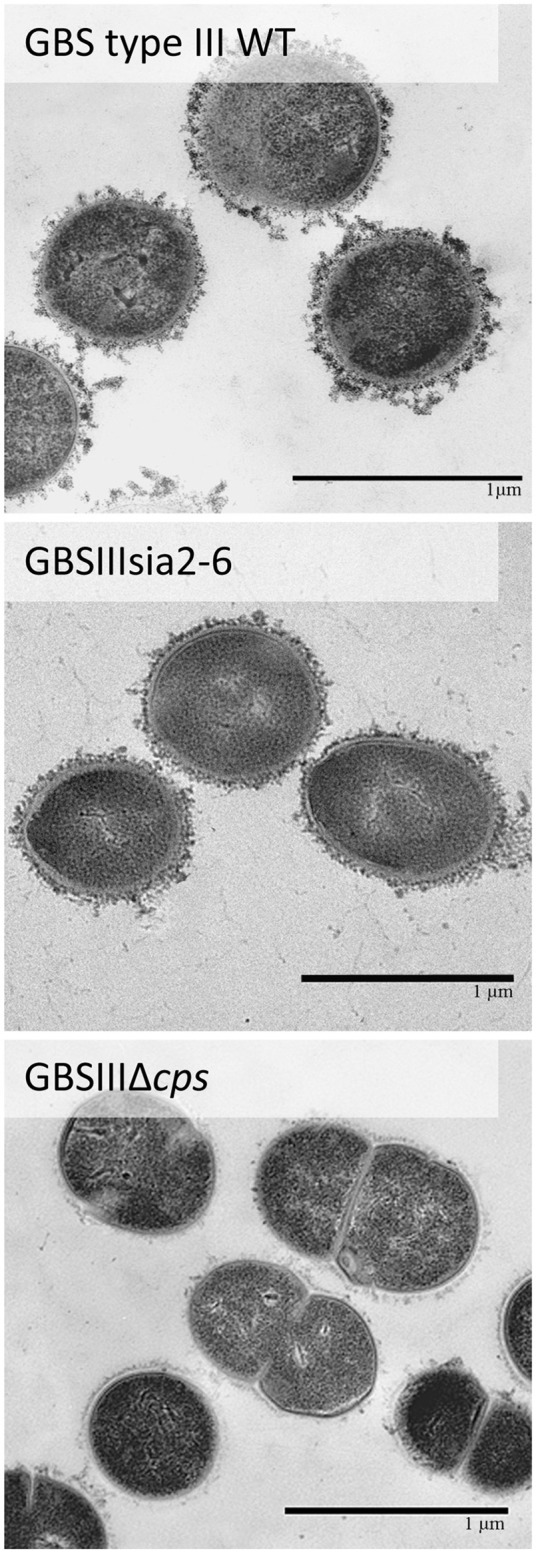
Transmission electron micrographs showing capsular polysaccharide (CPS) expression by GBS type III mutant carrying exogenous α-2,6-sialyltransferase. The CPS was labeled with polycationic ferritin. GBS type III wild-type strain was surrounded by a consistent CPS layer, whereas the GBSIIIsia2,6 (Δ*cps3K*/*cps2N*) mutant showed intermediate state of encapsulation. The non-encapsulated strain GBSIIIΔ*cps* is depicted as negative control. Bars = 1 μm.

Further investigation of CPS expression at the bacterial surface was done by TEM, which showed a slim CPS surrounding the bacteria for mutants GBSIIIsia2,6 (Figure 11) and GBSVsia2,6 (Figure 8), whereas a thicker CPS was observed for respective wild-type strains. In contrast, non-encapsulated control strains GBSIIIΔ*cps* (Figure 11) and GBSVΔ*cps* (Figure 8) showed complete absence of CPS expression. Purified CPS yield recovered from mutants GBSIIIsia2,6 and GBSVsia2,6 was significantly reduced when compared to wild-type strains (Table 2), confirming intermediate levels of encapsulation. Analyses of purified CPSs by SEC-MALS also showed reduced weight-average molecular mass (*M*_w_) for the two mutant strains derived CPSs (Table 2).

**Figure 12:**
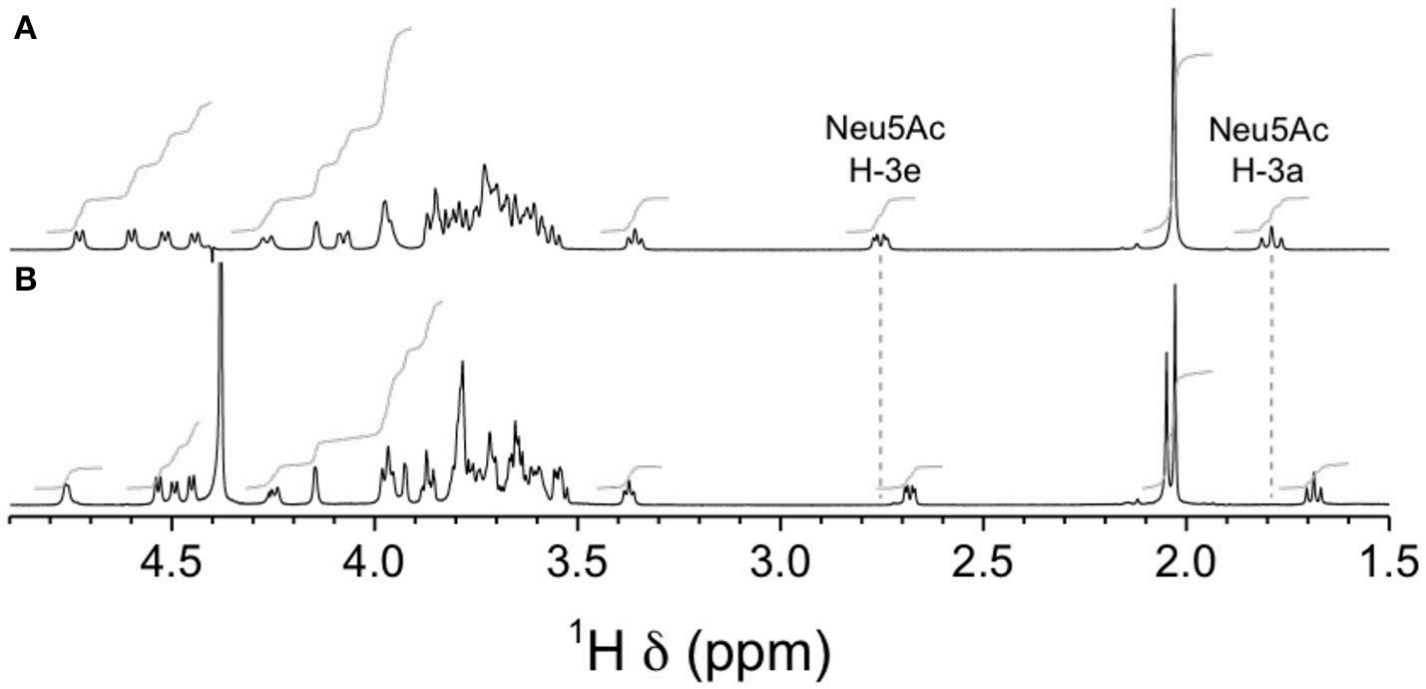
^1^H NMR spectra of native GBS type III and mutant GBSIIIsia2,6 capsular polysaccharide (CPSs). **(A)** Wild-type GBS type III, D_2_O, 500 MHz, 90° pulse with presaturation, 62°C. **(B)** GBSIIIsia2,6 (Δ*cps3K*/*cps2N*) mutant, 33 mM phosphate pD 8.0 in D_2_O, 700 MHz, 90° pulse, 65°C.

**Scheme 1:**
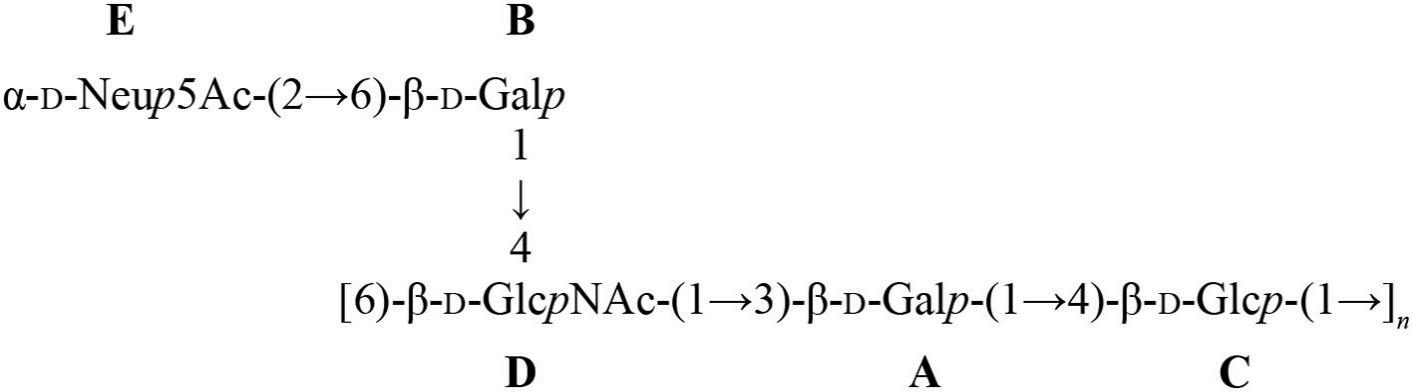
Structure of the GBSIIIsia2,6 mutant CPS with residue labels.

NMR analyses were used to confirm the nature of sialic acid linkage. Reporter resonance signals H-3e and H-3a were found at lower frequencies for the GBSIIIsia2,6 mutant compared to the wild-type GBS type III CPSs (Figure 12), consistent with sialic acid being 2,6- instead of 2,3-linked to the galactose residue (Machytka et al., 1994). Since major differences were also visible in other spectral regions, complete structural analysis was performed using a series of 1D and 2D experiments. Residues were labeled A–D in order of increasing chemical shift of their anomeric protons. On the COSY spectrum, correlations from anomeric protons could be followed up to A4, B4, C3, and D2 and from E3 protons to E4 (Supplementary Figure S1). This was extended on the TOCSY spectrum (not shown) up to C5, D5, and E6, which confirmed the *galacto* configuration for residues A and B and the *gluco* configuration for residues C and D. Intra-residue correlations between axial protons in positions 1, 3, and 5 were observed on the ROESY spectrum (not shown) for residues A–D. Reporter resonances were found on the ^13^C spectrum: 3 carbonyl, 5 anomeric (Supplementary Figure S2 trace), 2 amino (Supplementary Figure S2 trace), 1 methylene of sialic acid, and 2 acetyl methyl carbons. The DEPT spectrum (Supplementary Figure S2 trace) confirmed a linkage at position 6 for two sugar residues. Carbons were assigned using the HSQC (Supplementary Figure S2) and HSQC–TOCSY (not shown) spectra, which also allowed identifying previously unassigned proton resonances. Full ^1^H and ^13^C assignments are listed in Supplementary Table S2. When compared to corresponding methyl glycosides, ^13^C α glycosidation shifts of 2.32–9.62 ppm were observed for carbons A3, B6, C4, D4, and D6. On the ROESY spectrum (not shown), a correlation was observed between the *N*-acetyl CH_3_ at δ 2.05 and D2. In addition, a few inter-residue correlations could readily be identified: C4–6/A1, D6/C1, A2–3/D1. Inter-residue correlations found on the HMBC spectrum (not shown), both from anomeric carbons (A1/C4, B1/D4, C1/D6′, D1/A3, E2/B6, and E2/B6′) and to anomeric protons (C4/A1, D4/B1, D6/C1, and A3/D1), confirmed the true linkage positions for all residues. Finally, the experiment also allowed assignments of acetyl carbons. The structure is shown in Scheme 1.

Chemical shift differences between mutant and wild-type CPSs are reported in Supplementary Table S3. Significant differences (>1 standard deviation) were found for protons B1, B3, B6, B6′, D4, and E3a and for carbons B1, B2, B3, B5, B6, and D4. The transformation from 2,3- to 2,6-linked sialic acid was clearly evidenced by the large negative and positive ^13^C chemical shift differences for B3 and B6, respectively. The large ^1^H and ^13^C chemical shift differences at D4 indicate that not only the presence of sialic acid (Brisson et al., 1997), but also its linkage position exerts conformational control over the CPS backbone.

**Figure 13:**
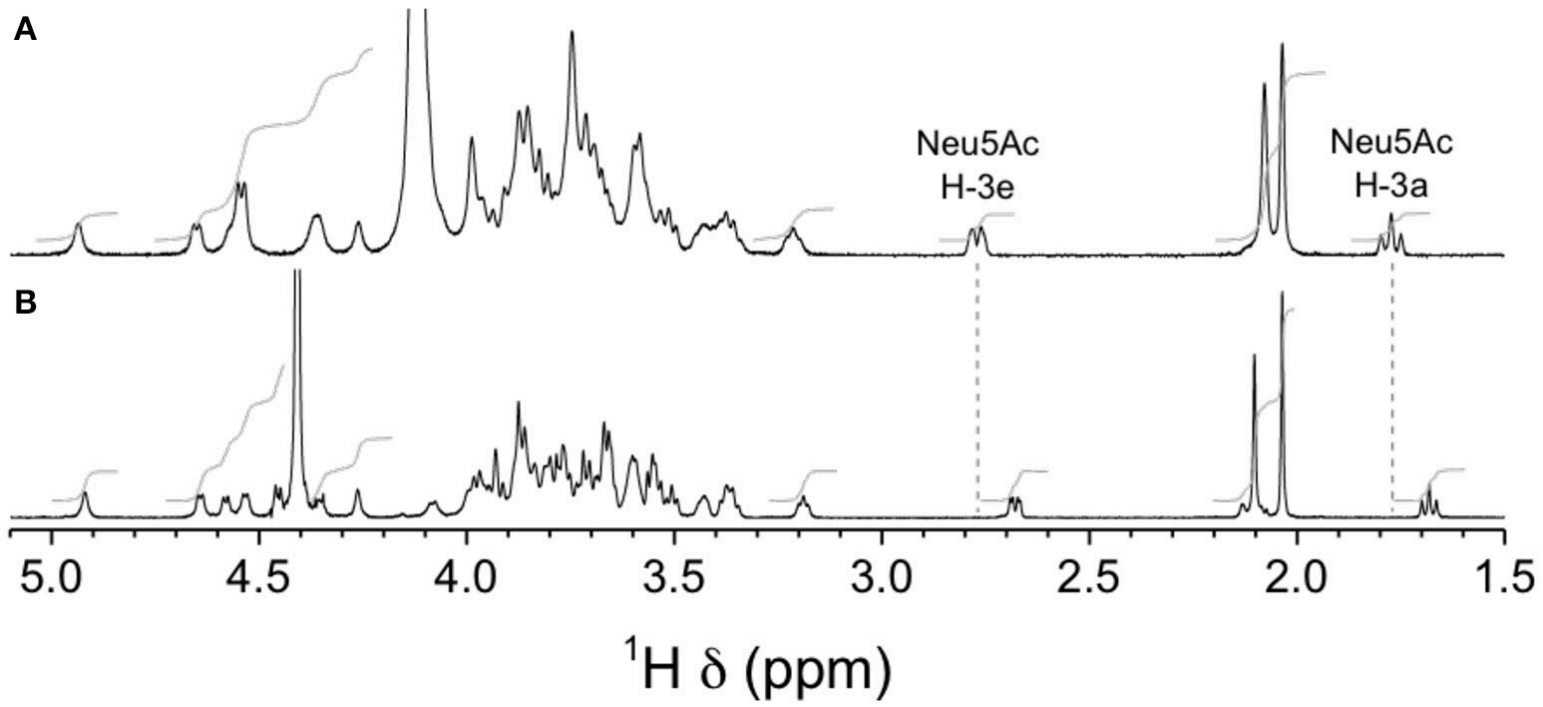
^1^H NMR spectra of native GBS type V and mutant GBSVsia2,6 capsular polysaccharides (CPSs). **(A)** Wild-type GBS type V, D_2_O, 500 MHz, 30° pulse, 80°C. **(B)** GBSVsia2,6 (Δ*cps5K/cps2N*) mutant, 33 mM phosphate pD 8.0 in D_2_O, 700 MHz, 30° pulse, 61°C.

**Scheme 2:**
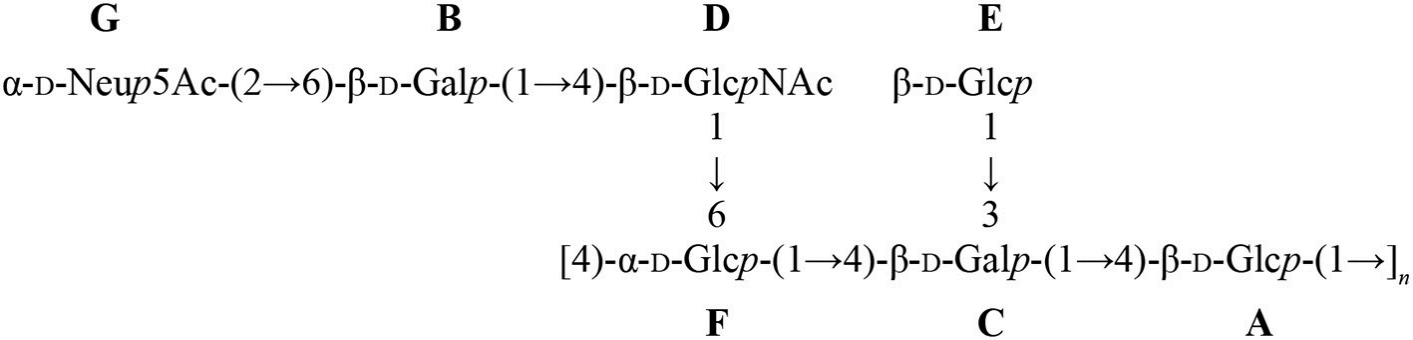
Structure of the GBSVsia2,6 mutant CPS with residue labels.

Similar differences in sialic acid ^1^H reporter resonance signal positions were observed for the GBSVsia2,6 mutant compared to the wild-type GBS type V CPS (Figure 13), confirming the transformation to a 2,6-linkage for sialic acid in this serotype as well. Complete structural analysis was also performed for this CPS. Residues were labeled A–F in order of increasing chemical shift of their anomeric protons. On the COSY spectrum, correlations from anomeric protons could be followed up to A3, B3, C4, D2, E6, E6′, and F2 and from G3 protons to G4 (Supplementary Figure S3). This was extended on the TOCSY spectrum (not shown) up to A4, B4, D5, F5, and G6, and the *galacto* configuration was confirmed for residues B and C and the *gluco* configuration for residue D. Intra-residue correlations between axial protons in positions 1, 3, and 5 were observed on the ROESY spectrum (not shown) for residues A–E. Reporter resonances were found on the ^13^C spectrum: 3 carbonyl, 7 anomeric (Supplementary Figure S4 trace), 2 amino (Supplementary Figure S4 trace), 1 methylene of sialic acid, and 2 acetyl methyl carbons. The DEPT spectrum (Supplementary Figure S4 trace) confirmed a linkage at position 6 for two sugar residues. Carbons were assigned using the HSQC (Supplementary Figure S4) and HSQC–TOCSY spectra (not shown), which also allowed identifying previously unassigned proton resonances. Full ^1^H and ^13^C assignments are listed in Supplementary Table S4. When compared to corresponding methyl glycosides, ^13^C α glycosidation shifts of 2.32–10.26 ppm were observed for carbons A4, B6, C3, C4, D4, F4, and F6. On the ROESY spectrum (not shown), a few inter-residue correlations could readily be identified: A1/F4, B1/D3–4, B1/D6′, C1/A3–4, E1/C3, and F1/C4–5. Inter-residue correlations found on the HMBC spectrum (not shown), both from anomeric carbons (B1/D4, C1/A4, G2/B6, and G2/B6′) and to anomeric protons (D4/B1, A4/C1, and C3/E1), confirmed the true linkage positions for several residues. Again, the experiment also allowed assignments of acetyl carbons. The structure is shown in Scheme 2.

Only anomeric proton chemical shifts have been reported for the wild-type type V CPS (Wessels et al., 1991), so most chemical shift differences, and as a consequence conformational control, cannot be evaluated in this case.

## Discussion

This study dissected for the first time the effect of sialic acid synthesis, sialic acid linkage, and sialyltransferase specificity on CPS expression by the only two so far described Gram-positive bacterial species able to sialylate their CPSs. We demonstrated that in spite of common CPS structural characteristics and similarities in the *cps* loci, sialic acid exerts differential control of CPS expression by *S. suis* and GBS.

The regulation of *S. suis* CPS synthesis is not clearly established. An interesting hypothesis is that *S. suis* may modulate the production of its CPS according to the environment. As a “wrapping” surface component, decreased CPS thickness would promote interaction of different surface adhesins with their ligands and consequently increase adhesion to host epithelial cells during colonization. Once in the bloodstream, optimal CPS expression at the bacterial surface is required to resist immune-clearance. Yet, there is no evidence that *S. suis* actually modulates CPS thickness *in vivo*. Nevertheless, some regulators or proteins are able to influence *in vitro* the expression of CPS synthesis genes, such as the regulator protein of carbon metabolism Ccpa (Willenborg et al., 2011), the regulator CovR (Pan et al., 2009), the global regulator CodY (Feng et al., 2016) and the di-adenosine monophosphate phosphodiesterase (Du et al., 2014). In addition, a study highlighting the role of a messenger RNA in the modulation of CPS synthesis has been recently published (Xiao et al., 2017). In fact, *S. suis* CPS regulation may be subjected to several factors and/or environmental conditions.

Compared to GBS, CPS structures and *cps* loci have been less studied in *S. suis*. To date, there was no clear information about *cps* locus transcription for *S. suis*. In order to investigate the presence of additional promoters within the *cps* locus of *S. suis*, RT-PCR analyses were applied and demonstrated that the *cps* locus is transcribed as a single polycistronic message, confirming that the *S. suis cps* locus is regulated from a single downstream promoter, as it was also described for GBS (Chaffin et al., 2000). Indeed, sialyltransferase, and sialic acid synthesis genes are under the same promoter than the glycosyltransferase and CPS regulatory proteins. Giving the fact that GBS and *S. suis* sialyltransferases are regulated in the same manner, substitution between their sialyltransferase genes was thus conceivable.

Firstly, we investigated the role of the sialyltransferase in CPS expression by *S. suis*. It is already known that mutations in genes encoding regulatory enzymes (*cpsB* and *cpsD*), glycosyltransferases (*cpsE* and *cpsF*) and sialic acid synthesis pathway (*neuC*) result in a non-encapsulated phenotype for *S. suis* (Smith et al., 1999; Lecours et al., 2012; Roy et al., 2015). Since several attempts in our laboratory to directly knockdown the sialyltransferase gene (*cps2N*) in the genome of *S. suis* serotype 2 failed due to mutation-induced lethality, confirming a previous report (Lakkitjaroen et al., 2014), we developed an indirect three-step mutagenesis approach based on double cross-over homologous recombination to delete *S. suis cps2N*. Indeed, mutations in side-chain formation (*cpsJ*), sialylation (*cpsN*), polymerase (*cpsL*), and flippase (*cpsO*) genes are lethal for *S. suis*, but also for other streptococci, as it is the case for *Streptococcus pneumoniae* (Xayarath and Yother, 2007; Lakkitjaroen et al., 2014). In contrast, some of these mutations are known to be non-lethal for GBS type III, as it is the case for mutations in the sialyltransferase gene (*cpsK*) (Chaffin et al., 2005), suggesting differences in CPS synthesis between *S. suis* and GBS. The lethality of these mutations in *S. suis* and *S. pneumoniae* is hypothesized to be due to the sequestration of undecaprenyl-phosphate carrier CPS precursor in the incomplete CPS synthesis pathway that is also needed for other biological functions, such as synthesis of cell-wall peptidoglycan. However, a mutation in the *S. suis cpsN* gene can occur in presence of suppressor mutations in other CPS genes that inhibit CPS synthesis, as it was demonstrated with suppressor mutation in *cpsEF* (Lakkitjaroen et al., 2014). Giving the fact that deletion of the *neuC* gene also inhibits CPS synthesis (Lecours et al., 2012), we used it as suppressor mutation in order to indirectly knockdown *cps2N*. Unfortunately, *S. suis* type 2 sialyltransferase gene deletion (SS2ΔsiaT) resulted in a non-encapsulated phenotype, confirming the critical role of sialic acid in the CPS expression by *S. suis*. Giving the fact that complementation of SS2ΔsiaT mutant restored CPS synthesis and no mutation was found in *cps2E* and *cps2F* genes (data not shown), the resulting phenotype is most likely caused by the deletion of *cps2N*. Based on these results, one possible hypothesis is that the polymerase and/or the flippase enzymes of *S. suis* recognize the sialic acid moiety in the polysaccharide repeating unit in order to polymerize and/or export the CPS, respectively.

In contrast to *S. suis*, the presence of sialic acid in the polysaccharide repeating unit is not absolutely required for CPS expression by GBS, as sialyltransferase or sialic acid synthase gene deletion mutants of GBS type III (Chaffin et al., 2005; Lecours et al., 2012) and GBS type V (this work) are still able to express CPS at their surface. Yet, the obtained asialo encapsulated mutants present reduced CPS amounts, suggesting that the sialic acid pathway is important, albeit not vital, for optimal CPS expression by GBS types III and V. The herein-observed low-encapsulation phenotype of GBSVΔsiaT and GBSVΔsynth mutants may result of reduced efficiency of the polymerase to polymerize the polysaccharides in absence of sialic acid. Indeed, SEC-MALS analyses of purified CPSs of mutants GBSVΔsiaT and GBSVΔsynth confirmed reduced CPS *M*_w_ compared to the wild-type CPS, suggesting that sialic acid is necessary for optimal CPS polymerization. This is in contrast to results reported by Chaffin et al. (2005), where the GBS type III asialo mutant (Δ*cpsK*) shows longer polysaccharide chain length than the wild-type sialyled CPS. Indeed, for GBS type III, reduced CPS amounts in the asialo mutant (Δ*cpsK*) do not seem to be related to shorter polysaccharide chains, but is likely due to reduced transfer of CPS precursors across the cytoplasmic membrane (Chaffin et al., 2005). Our results with GBSVΔsiaT and GBSVΔsynth mutants indicate that differences exist between these two serotypes in how they behave in terms of CPS polymerization and export in the absence of sialic acid. In spite of these inter-serotype differences, when compared to *S. suis*, overall GBS polymerase and/or flippase seem to be more versatile and less specific for substrate than the *S. suis* respective enzymes.

To evaluate how the specificity of the sialyltransferase and the resulting sialic acid linkage affect CPS expression, we performed for the first time inter-species sialyltransferase exchange between these two Gram-positive bacterial species. Since aforementioned differences between GBS types III and V, the two serotypes were analyzed and compared to two different serotypes (2 and 14) of *S. suis*. Substitution of the *S. suis* α-2,6-sialyltransferase by the GBS α-2,3-sialyltransferase in *S. suis* serotypes 2 and 14 also results in a non-encapsulated phenotype. This phenotype might be related to a high specificity of *S. suis* polymerase and/or flippase for α-2,6 terminal sialic acid in both *S. suis* serotypes. It can be hypothesized that *S. suis* polysaccharide chains need to be α-2,6-sialylated in order to be recognized by *S. suis* polymerase and/or flippase enzymes. Another hypothesis is that the GBS α-2,3 sialyltransferases are specific to GBS polysaccharide structures and thus unable to recognize *S. suis* polysaccharide chain/structure in order to transfer sialic acid to the terminal galactose, consequently inhibiting polymerization and/or exportation of the CPS. In fact, GBS type III and *S. suis* sialyltransferases share only 33% of protein identity. However, the sialyltransferase gene is 100% identical between *S. suis* serotypes 2 (strain P1/7) and 14 (strain DAN13730) and highly conserved among other sialylated *S. suis* serotypes, such as serotypes 1 and 1/2, suggesting that the *S. suis* sialyltransferase may recognize a common epitope/component (Okura et al., 2013). In contrast, GBS sialyltransferases of types III and V express significant differences in the 5′ region of the *cpsK* gene and may recognize different epitopes/components (Chaffin et al., 2005). In this study, we used the GBS type III sialyltransferase in *S. suis*, thus we cannot rule out the possibility that the GBS type V sialyltransferase may be able to sialylate *S. suis* polysaccharides due to the differences between type III and V sialyltransferases (Chaffin et al., 2005).

In contrast to *S. suis*, GBS type III and V mutants carrying the α-2,6-sialyltransferase of *S. suis* are still able to express CPS at the bacterial surface, albeit at a reduced amount. These results support the hypothesis that GBS polymerase and/or flippase seem to be more versatile than those of *S. suis*. As such, modification of sialic acid linkage (α-2,3 to α-2,6) affects, but not completely inhibits, recognition of polysaccharide subunits/chain by polymerase and/or flippase and consequently leads to a CPS of reduced thickness and a diminished polysaccharide chain length (represented by *M*_w_) in both mutants. Importantly, we demonstrated by 1D and 2D NMR spectroscopy that the overall CPS structure of GBS types III and V was preserved in GBSIIIsia2,6 and GBSVsia2,6 mutants, respectively, and that only the linkage between sialic acid and side-chain galactose was effectively changed from α-2,3 to α-2,6 as expected. The *S. suis* serotype 2 α-2,6-sialyltransferase probably recognizes common pattern(s) shared by both *S. suis* and GBS in CPS components/structure. This is the first time that exogenous sialyltransferase replacement is used in order to express CPSs with modified sialic acid linkage in Gram-positive bacteria.

The nature of carbohydrate epitopes, such as those present in bacterial CPSs, is diverse and can be linear or conformational. Serological analyses have suggested that the side chain, and more particularly the terminal sialic acid, constitutes one important epitope for *S. suis* serotype 2 (Van Calsteren et al., 2016). Nevertheless, the α-2,6 substitution in GBS type III and V CPSs failed to confer immunological cross-reaction with anti-*S. suis* type 2 or type 14 CPS antibodies (unpublished observations), highlighting the complexity of these carbohydrate epitopes.

In conclusion, a critical role of sialic acid (and its linkage) in *S. suis* CPS expression at the bacterial surface was demonstrated. Unfortunately, the non-encapsulated phenotype makes impossible so far to study the precise role of sialic acid in *S. suis* pathogenesis. In contrast, GBS is able to express asialo CPS or α-2,6-sialylated CPS, although the amount of polysaccharide at the bacterial surface is reduced. Albeit this limited expression of CPS at the bacterial surface might compromise accurate studies on the role of sialic acid linkage in host-pathogen interactions, the modified GBS type III and V CPSs represent new tools to study CPS immunogenicity and biochemistry.

## Author contributions

MS, DT, MO, and DR: Designed research studies; DT and MO: Provided plasmids and guidance for mutagenesis studies; DR and AD: Performed research experiments; GG-D: Contributed to CPS purification and analysis; M-RC: performed NMR analysis; DR, M-RC, MS, and MG: Analyzed the data; DR and M-RC: Wrote the first draft of the manuscript. All authors reviewed and approved the manuscript.

### Conflict of interest statement

The authors declare that the research was conducted in the absence of any commercial or financial relationships that could be construed as a potential conflict of interest.

## Abbreviations

CPS: capsular polysaccharide
ELLA: enzyme-linked lectin assay
GBS: Group B *Streptococcus*
MAL-I: *Maackia amurensis* leukoagglutinin
*M*_w_: weight-average molecular mass
NMR: nuclear magnetic resonance
SEC-MALS: size-exclusion chromatography coupled with multi-angle light scattering
SNA-I: *Sambucus nigra* agglutinin
Sp: spectinomycin
TEM: transmission electron microscopy
THA: Todd-Hewitt agar
THB: Todd-Hewitt broth.
