# Supplementary material for: Capsular Sialyltransferase Specificity Mediates Different Phenotypes in Streptococcus suis and Group B Streptococcus
Source: Front Microbiol. 2018 Apr 3;9:545. doi: 10.3389/fmicb.2018.00545 (PMC5891629; doi:10.3389/fmicb.2018.00545)
Supplement: Supplementary file 1 [file Presentation1.pdf]

Supplementary Material

Capsular Sialyltransferase Specificity Mediates Different Phenotypes in *Streptococcus suis* and Group B *Streptococcus*

David Roy, Daisuke Takamatsu, Masatoshi Okura, Guillaume Goyette-Desjardins, Marie-Rose Van Calsteren, Audrey Dumesnil, Marcelo Gottschalk and Mariela Segura*

**** Correspondence:***
Mariela Segura
mariela.segura@umontreal.ca

Supplementary Figures and Tables:

Figure S1. NMR COSY spectrum of the GBSIIIsia2,6 mutant CPS.
Figure S2. NMR HSQC spectrum of the GBSIIIsia2,6 mutant CPS.
Figure S3. NMR COSY spectrum of the GBSVsia2,6 mutant CPS.
Figure S4. NMR HSQC spectrum of the GBSVsia2,6 mutant CPS.
Table S1. Oligonucleotide primers used in this study.
Table S2. NMR chemical shifts of the GBSIIIsia2,6 mutant CPS.
Table S3. NMR chemical shift differences between the GBSIIIsia2,6 mutant and wild-type GBS type III CPSs.
Table S4. NMR chemical shifts of the GBSVsia2,6 mutant CPS.

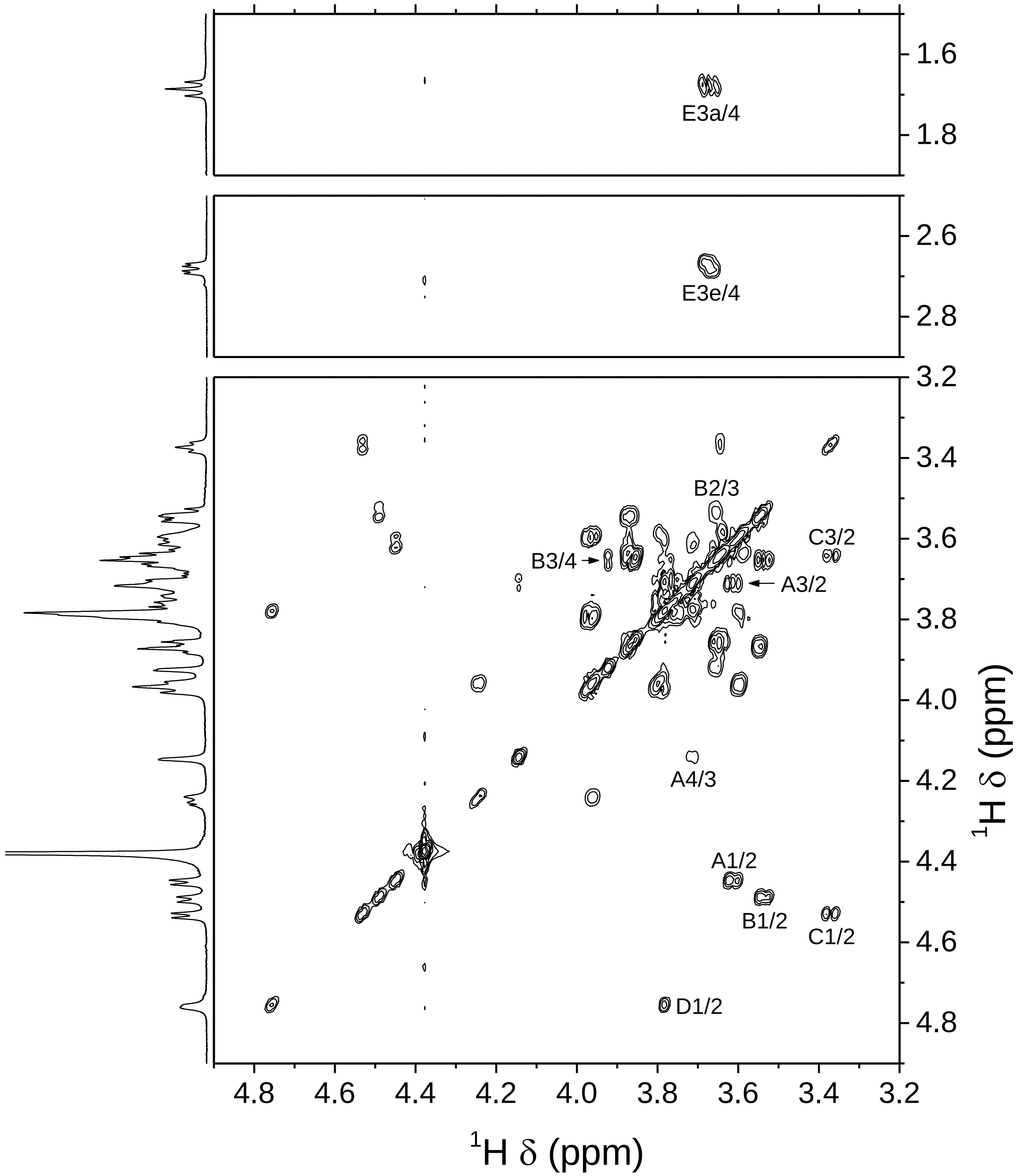

Supplementary Figure S1. Portions of the 700-MHz 2D NMR COSY spectrum of the GBSIIIsia2,6 mutant CPS in 33 mM phosphate pD 8.0 in D_2O at 65°C. 512 increments of 2 K complex data points were acquired in magnitude mode with a digital resolution of 3.4 Hz/point in the t_2 dimension and 13.7 Hz/point in the t_1 dimension. The t_2 dimension was processed by multiplication with an unshifted sine bell window function and Fourier transform, and the t_1 dimension was processed by Zhu-Bax linear prediction to 1024 points, multiplication with an unshifted sine bell window function, Fourier transform, and magnitude calculation. The f_1 trace corresponds to the 1D ^{1}H spectrum (see Figure 12B).

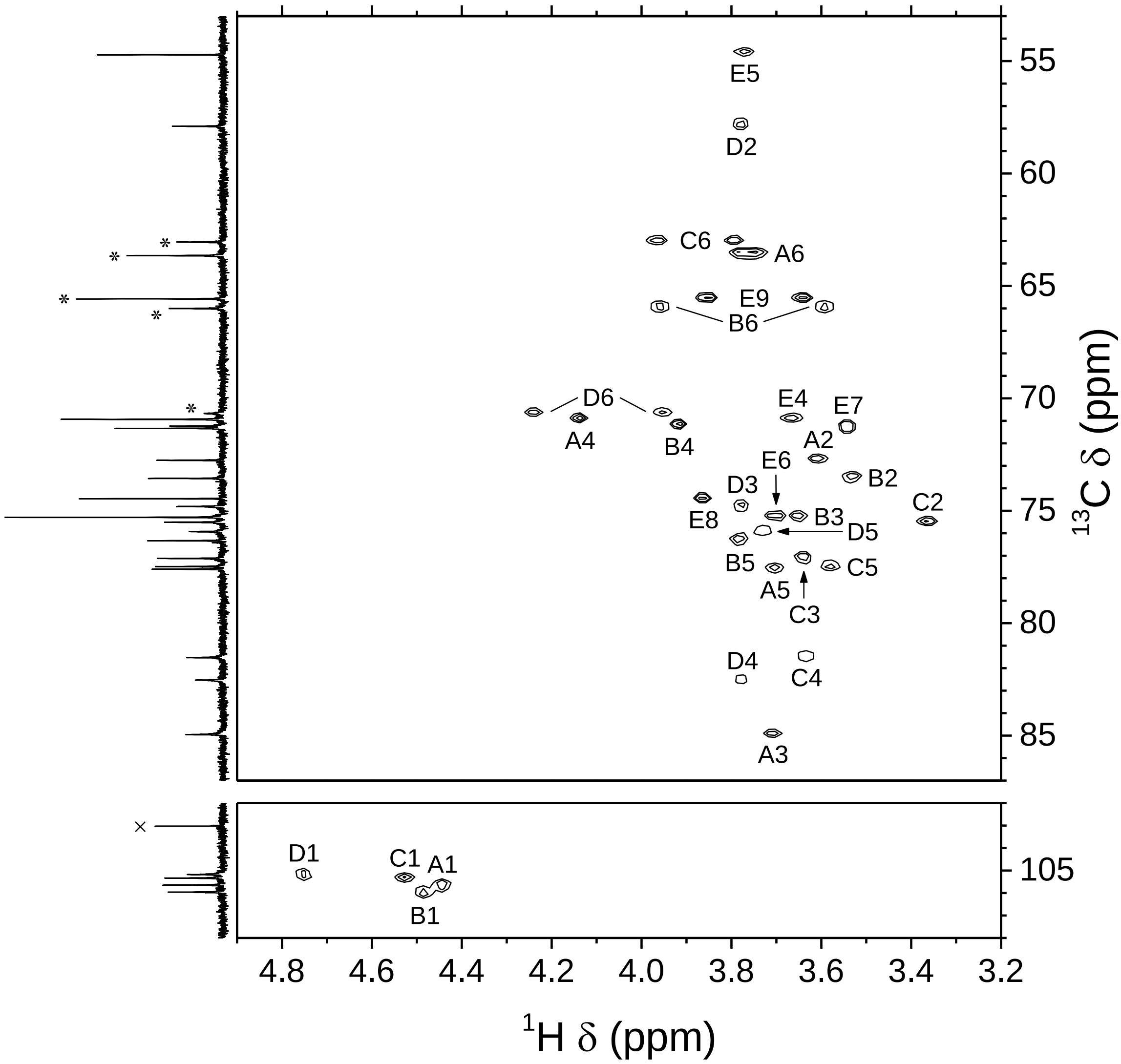

Supplementary Figure S2. Portions of the 176-MHz ge-2D NMR HSQC spectrum of the GBSIIIsia2,6 mutant CPS in 33 mM phosphate pD 8.0 in D_2O at 65°C. 2 × 200 increments of 512 complex data points were acquired in the echo–antiecho mode with a digital resolution of 9.8 Hz/point in the t_2 dimension and 57.3 Hz/point in the t_1 dimension. The t_2 dimension was processed by multiplication with a π/2 shifted sine bell window function, Fourier transform, and phase correction, and the t_1 dimension was processed by Zhu-Bax linear prediction to 400 points, multiplication with a π/2 shifted sine bell window function, zero filling, Fourier transform, and phase correction. Only positive contours are shown. The f_1 trace corresponds to the 1D ^{13}C spectrum. *, signals inverted on the DEPT spectrum; ×, signal absent on the DEPT spectrum.

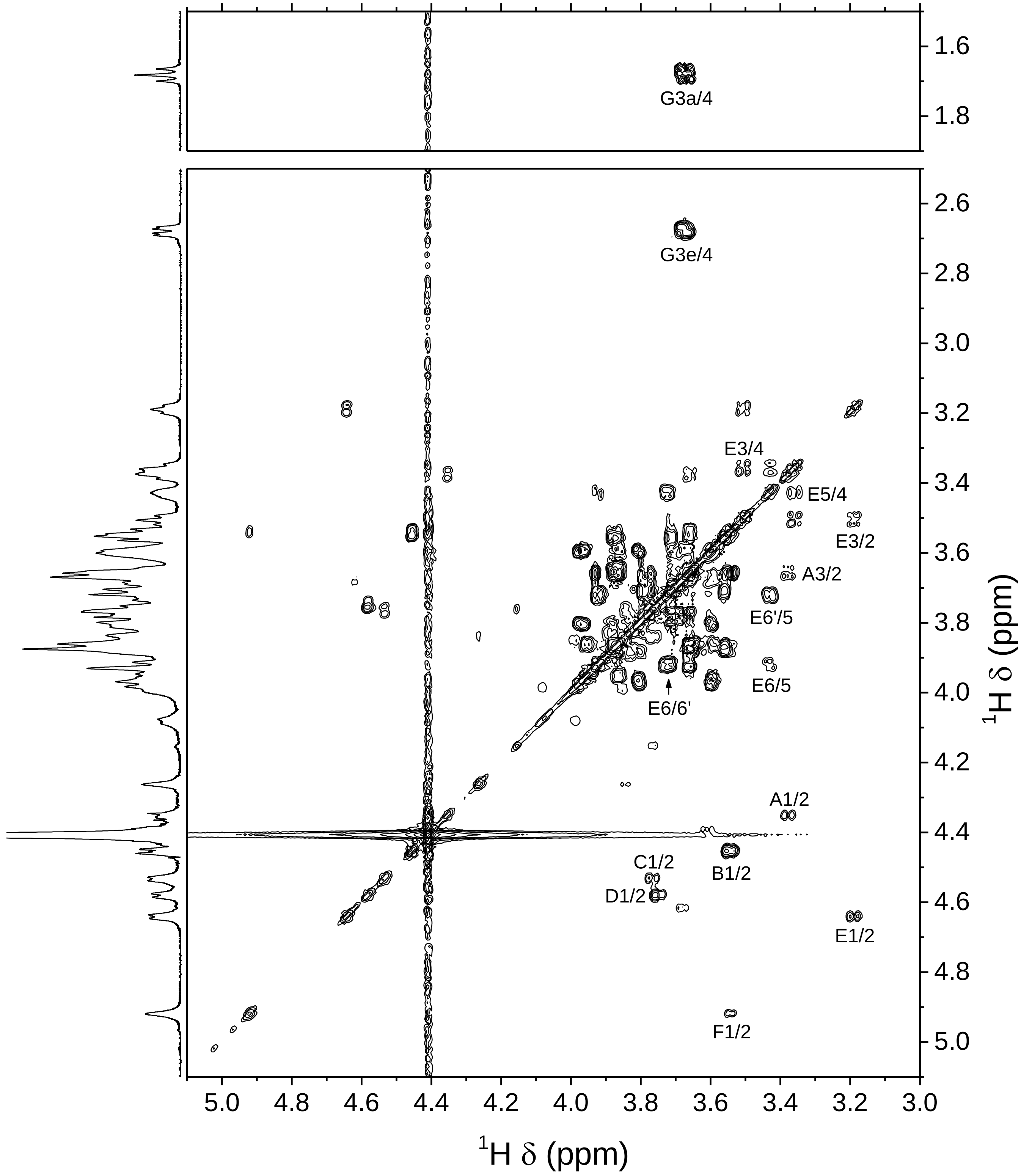

Supplementary Figure S3. Portions of the 700-MHz 2D NMR COSY spectrum of the GBSVsia2,6 mutant CPS in 33 mM phosphate pD 8.0 in D_2O at 61°C. 512 increments of 1 K complex data points were acquired in magnitude mode with a digital resolution of 4.2 Hz/point in the t_2 dimension and 8.3 Hz/point in the t_1 dimension. Processing was as for Supplementary Figure S1. The f_1 trace corresponds to the 1D ^{1}H spectrum (see Figure 13B).

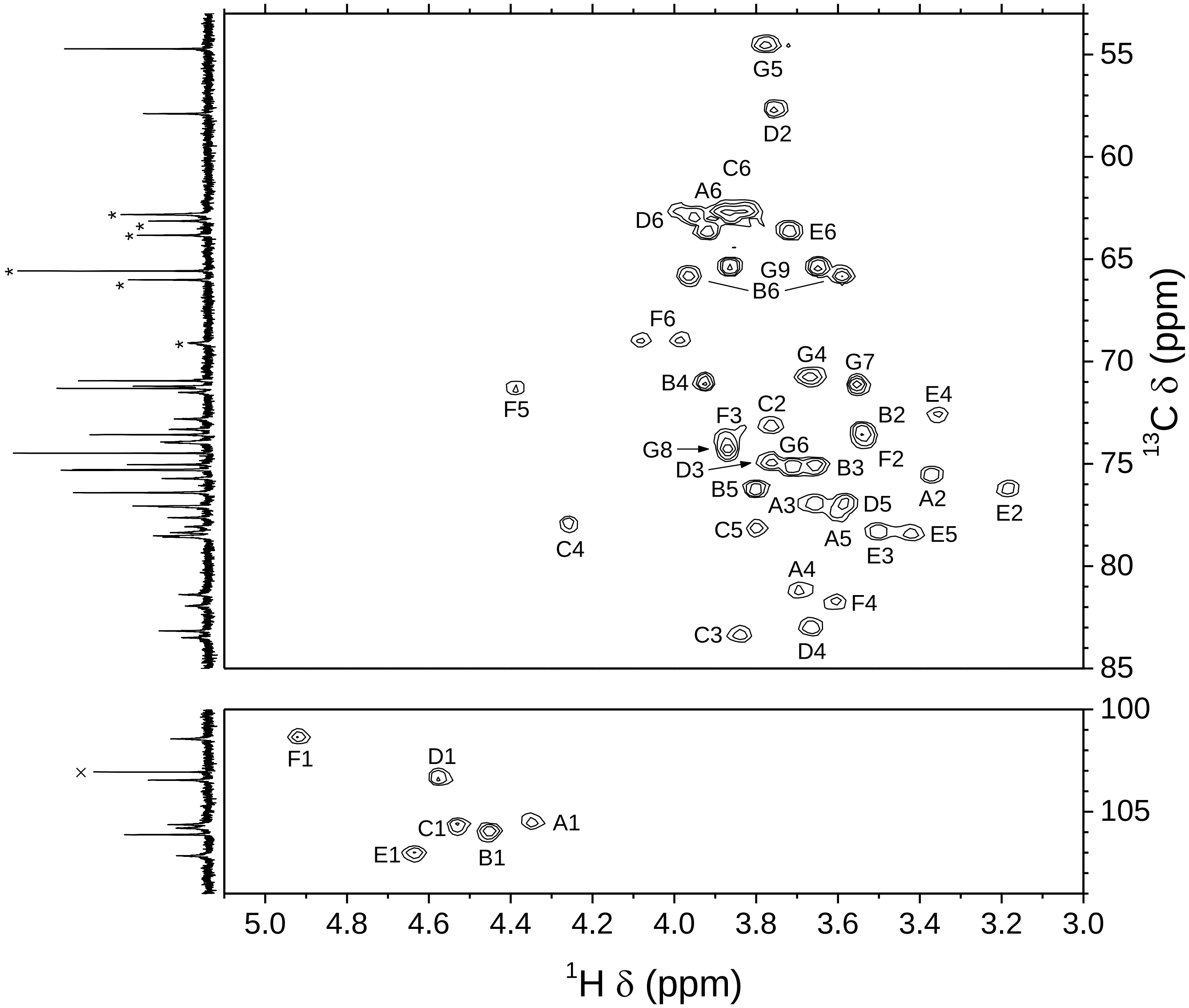

Supplementary Figure S4. Portions of the 176-MHz ge-2D NMR HSQC spectrum of the GBSVsia2,6 mutant CPS in 33 mM phosphate pD 8.0 in D_2O at 61°C. 2 × 200 increments of 425 complex data points were acquired in the echo–antiecho mode with a digital resolution of 10.0 Hz/point in the t_2 dimension and 79.1 Hz/point in the t_1 dimension. Processing was as for Supplementary Figure S2. Only positive contours are shown. The f_1 trace corresponds to the 1D ^{13}C spectrum. *, signals inverted on the DEPT spectrum; ×, signal absent on the DEPT spectrum.

SUPPLEMENTARY TABLE S1. Oligonucleotide primers used in this study.

Oligonucleotide primers, sequence (5' – 3')[a]		Constructs or RT-PCR reactions
cps2_ID1	CGTTGAATTTGTGGAACGGC	p4sia2,3_2
cps2_ID2	AGTTGCTCCCTGACATCTGG	p4sia2,3_2
cps2_ID3	GTAGCAGGTCTTGCCCCTTATC	p4sia2,3_2
cps2_ID4	GCAAGTGTGTAGCCGAAACTG	p4sia2,3_2
cps2_ID7	CCCAGTATCCCCCTTTATTTTC	p4sia2,3_2
cps2_ID8	CCAACAATTCGTGGTTCTCCTTCTACATAC	p4sia2,3_2
cps2_ID9	GGTGCAAGGATTAGCCGAAATGGCAGGTAG	p4sia2,3_2
cps2_ID10	CCATTACACGAGCGATGAAATC	p4sia2,3_2
cpsK_5	TTTATCAGCATTAGACGAGCG	p4sia2,3_2/14
cpsK_6	CCGGGCTGAACTTAAAGAACC	p4sia2,3_2/14
cpsK_11	GTATGTAGAAGGAGAACCACGAATTGTTGG	p4sia2,3_2/14
cpsK_12	CTACCTGCCATTTCGGCTAATCCTTGCACC	p4sia2,3_2/14
sia14_IDI	CGTTGAATTTGTGGAACGGC	p4sia2,3_14
sia14_ID2	AGTTGCTCCCTGACATCTGG	p4sia2,3_14
sia14_ID3	GTAGCAGGTCTTGCCCCTTATC	p4sia2,3_14
sia14_ID4	GCAAGTGTGTAGCCGAAACTG	p4sia2,3_14
sia14_ID7	CCCAGTATCCCCCTTTATTTTC	p4sia2,3_14
sia14_ID8	CCAACAATTCGTGGTTCTCCTTCTACATAC	p4sia2,3_14
sia14_ID9	GGTGCAAGGATTAGCCGAAATGGCAGGTAG	p4sia2,3_14
sia14_ID10	CCATTACACGAGCGATGAAATC	p4sia2,3_14
cpsJ_ID1	TGGTTCGACGGATAATTGTGC	p4sia2,6_III
cpsJ_ID2	CTGCTCCCGATAAGCAAACTC	p4sia2,6_III
cpsL_ID3	GTTGACACAGCCACTTGCAC	p4sia2,6_III
cpsL_ID4	CACCAGCAGCTAATAATGTCCC	p4sia2,6_III
cpsJ_ID7	GGGTTGTCAGAAGCTAGAAAC	p4sia2,6_III
cpsJ_ID8	TCTTGCCCCTTCATCACCAACAATTCGTGGT	p4sia2,6_III
cpsL_ID9	GTTGTCTCAGGTCTCCATTCGTCCACTGCG	p4sia2,6_III
cpsL_ID10	CAGACACAGTGACAATGAACCG	p4sia2,6_III
cps2L_ID5	TCTTCTGCAAGTCACCTCACC	p4sia2,6_III
cps2L_ID6	ACGACCCAATCAGGCAAACC	p4sia2,6_III
cps2L_ID11	ACCACGAATTGTTGGTGATGAAGGGCAAGA	p4sia2,6_III
cps2L_ID12	CGCAGTGGACGAATGGAGACCTGAGACAAC	p4sia2,6_III
sia5_ID13	TGCAGTGGTGTATTTTAGCG	p4sia2,6_V
sia5_ID23	TCCGTCCTTATTCCCTGTTC	p4sia2,6_V
sia5_ID33	GCCGGTAGAGCTATTACCATC	p4sia2,6_V
sia5_ID43	TAACCAATTTACACCAGCAGC	p4sia2,6_V
sia5_ID73	AGGATTAGTGTGAAGGAGAAGG	p4sia2,6_V
5V_8	TTGCCCTTCATCATTGACACACAAAATTAT	p4sia2,6_V
5V_92	GATTCATGTCTAAAATGGGATAACACATTC	p4sia2,6_V
sia5_ID103	GTCTCCTCCCATTATTTGAGC	p4sia2,6_V
5V_11	ATAATTTTGTGTGTCAATGATGAAGGGCAA	p4sia2,6_V
5V_122	GAATGTGTTATCCCATTTTAGACATGAATC	p4sia2,6_V
cps2L3_ID1	GGGAGTTGGGAGTTACTATG	p4Δ*cps2N*

cps2L3_ID2	CTGACATCTGGAAAATGCC	p4Δ*cps2N*
cps2L3_ID3	AATGGCAGGTAGTATCCG	p4Δ*cps2N*
cps2L3_ID4	GACCGTTTTTCCCTGAATG	p4Δ*cps2N*
cps2L3_ID5	GGTAGATACTTTCATTGCGACC	p4Δ*cps2N*
cps2L3_ID6	CGTGAGGGGATAGAACAAGGAAATCAGGAT	p4Δ*cps2N*
cps2L3_ID7	ATCCTGATTTCCTTGTTCTATCCCCTCACG	p4Δ*cps2N*
cps2L3_ID8	GCAACAACAGATAGGAAGC	p4Δ*cps2N*
Neu14C_ID5	TCTCAGCTCGAAATGACTCGTC	p4*neuC*
Neu14C_ID8	AGGTCCCTGACTCCGTCAAC	p4*neuC*
Neu5B_ID1	TTATTGGTCTTCAGACGAGCGG	p4Δ*neu5B*
Neu5B_ID2	GCATCAACACCACAAGACACG	p4Δ*neu5B*
Neu5B_ID3	ATATTACGGTGAAGCGCCCAGG	p4Δ*neu5B*
Neu5B_ID4	GAGGAGGTTTCGACTGGTACAC	p4Δ*neu5B*
Neu5B_ID5	TTCGGTTCATTGTCACTGTGTC	p4Δ*neu5B*
Neu5B_ID6	GCGTGAATCACGAATGCAACCAATCTCTGC	p4Δ*neu5B*
Neu5B_ID7	GCAGAGATTGGTTGCATTCGTGATTCACGC	p4Δ*neu5B*
Neu5B_ID8	AGTACCGCTTTCATCTGCTCTC	p4Δ*neu5B*
cps5K_ID1	AGGATTAGTGTGAAGGAGAAGG	p4Δ*cps5K*
cps5K_ID2	TTTGTAACTGCTCCCGATAAGC	p4Δ*cps5K*
cps5K_ID3	TTTAGTGGGGCTACCTCATGAC	p4Δ*cps5K*
cps5K_ID4	AGCGCCATAGGCTGCATAATG	p4Δ*cps5K*
cps5K_ID5	GAAGATGCAATCGAGAGAATGG	p4Δ*cps5K*
cps5K_ID6	CGCGGTGGACGAATGCGTGATAGTGTCACA	p4Δ*cps5K*
cps5K_ID7	TGTGACACTATCACGCATTCGTCCACCGCG	p4Δ*cps5K*
cps5K_ID8	TCCAAGCGAATAACCGAAAC	p4Δ*cps5K*
cpsN_pstI_F	GCGCCTGCAGTATCGAAGCTGTACAGGG	pMX*cpsN*
cpsN_EcoRI_R	CGCGGAATTCCGAGACCTGAGACAACTATTG	pMX*cpsN*
M2000F	GATAGTTTGTCAGCCAGTGG	RT-PCR (R1)
cpsA_R	GCCAATACTGCCACTCCTAC	RT-PCR (R1)
M1000F	CTGATTGCACCGATTCGGAG	RT-PCR (R3)
cpsA_R	GCCAATACTGCCACTCCTAC	RT-PCR (R3)
M500F	CAATTCTGCCAATCCCTCTTG	RT-PCR (R4)
cpsA_R	GCCAATACTGCCACTCCTAC	RT-PCR (R4)
cpsA_F	GTCAAGCGATGGTGTTCAAC	RT-PCR (R5)
cpsB_R	CACCGGCTTCAACATTTTG	RT-PCR (R5)
cpsB_F	GCGGCACGTATTGCAAATAG	RT-PCR (R6)
cpsD_R	GCACGAGCCATTAAGTGTTG	RT-PCR (R6)
cpsD_F	GGCAGTAGCAGAAGTTTATCC	RT-PCR (R7)
cpsF_R	GTAGCCATTCATGACCGTC	RT-PCR (R7)
cpsF_F	GTTGGACGATTTGTGCCTG	RT-PCR (R8)
cpsG_R	ACAGCCTGTGAAACTGTCAC	RT-PCR (R8)
cpsF3_F	TTTTCATGGTCACGAGGTTG	RT-PCR (R9)
cpsH_R	GTGTGATGTCCCATCGATAGC	RT-PCR (R9)
cpsH_F	GCGAGAGAAGCCTCTTATTC	RT-PCR (R10)
cpsJ_R	CATTTCCTAAGTCTCGCACC	RT-PCR (R10)
cpsJ_F	GATAGTGATTTGTCGGGAGGG	RT-PCR (R11)
cpsK_R	GCCATAATTACGGGCATCTG	RT-PCR (R11)
cpsK_F	CGCCAAGGGTGACTACTTAG	RT-PCR (R12)
cpsN_R	CGAGCGACAGATCATTGACC	RT-PCR (R12)
cpsN_F	GCTCGTACAATTTGACGAGG	RT-PCR (R13 and R14)
cpsO_R	CAGGCAAACCAAATAGGAGC	RT-PCR (R13)
neu1_R	ATCGGTCGTTCCATTTTCCTG	RT-PCR (R14 and R15)
cpsO_F	CGCTCCTATTTGGTTTGCC	RT-PCR (R15)
neu2_F	GGAAAAAATTGGTCGTCAAGC	RT-PCR (R16)
neu2_R	TGGCAACTGGTAGCATCTC	RT-PCR (R16)
neu3_F	CCTTGACAGAGCAACTCAC	RT-PCR (R17)

neu3_R	ACCAAGGAAGGACACCATC	RT-PCR (R17)
neu4_F	GGCCTCAACCATGAAAGAG	RT-PCR (R18)
neu4_R	GTTGTAAAATCTGTCGCCAAG	RT-PCR (R18)
neu5_F	TTTTCCATACCATTCGAGCTG	RT-PCR (R19)
neu5_R	TGCTCTGCTTTGGAAACAAG	RT-PCR (R19)
neu6_F	ATCGGTGGGATGACAGCTTC	RT-PCR (R21, R22, R23, R24 and R25)
neu6_R	TCCTTTTCACGACCTGACTTG	RT-PCR (R21)
neu6R_R	TGGTCAAACTTGTCAAAATC	RT-PCR (R22)
neu8R_R	TACTTGTTCGGAAGTTAGAGCC	RT-PCR (R23)

[a] *Oligonucleotide primers were from IDT (Coralville, IA); restriction sites are underlined.*

SUPPLEMENTARY TABLE S2. ^{1}H and ^{13}C NMR chemical shifts of the GBSIIIsia2,6 mutant CPS[a].

**Residue**		**1**		**2**	**3**		**4**	**5**	**6**		**7**	**8**	**9**		**CH_3**	**CO**
A	→3)-β-D-Gal-(1→	4.45	(7.7)[b]	3.62	3.72		4.15	3.71	3.79	3.76						
		105.65		72.76	84.96		70.93	77.60	63.65							
B	→6)-β-D-Gal-(1→	4.49	(8.8)	3.54	3.66		3.93	3.79	3.96	3.60						
		105.96		73.56	75.29		71.24	76.33	66.01							
C	→4)-β-D-Glc-(1→	4.53	(7.8)	3.38	3.65		3.64	3.59	3.97	3.80						
		105.34		75.51	77.12		81.53	77.48	63.05							
D	→3,6)-β-D-GlcNAc-(1→	4.76		3.78	3.78		3.78	3.74	4.24	3.96					2.05	
		105.18		57.90	74.81		82.53	75.93	70.67						25.07	177.45
E	α-D-Neu5Ac-(2→				2.68	1.69	3.67	3.78	3.70		3.55	3.87	3.86	3.65	2.03	
		175.97		103.03	42.82		71.34	54.72	75.29		70.93	74.46	65.58		24.77	177.64

[a] *Chemical shifts (^{1}H, top; ^{13}C, bottom) at 65°C in 33 mmol/L phosphate buffer pD 8.0 in D_2O in ppm referenced to internal DSS. ^{1}H chemical shifts were obtained from the 1D, COSY, TOCSY, or HSQC spectrum. ^{13}C chemical shifts were obtained from the 1D spectrum.*

[b] *Coupling constants ($J_{H\text{-}1\text{–}H\text{-}2}$) in parentheses.*

SUPPLEMENTARY TABLE S3. ^{1}H and ^{13}C NMR chemical shift differences between the GBSIIIsia2,6 mutant and wild-type GBS type III CPSs[a].

Residue	1	2	3		4	5	6		7	8	9	
A	0.01	0.02	0.00		−0.03	−0.01	−0.01	0.01				
	−0.20	0.01	−0.29		−0.09	−0.11	−0.15					
B	−0.14	−0.04	−0.45		−0.05	0.09	0.18	−0.15				
	1.16	1.40	−3.23		0.88	−1.45	2.32					
C	−0.02	0.01	−0.03		−0.04	−0.09	−0.04	−0.02				
	−0.03	0.16	0.07		0.37	0.02	0.26					
D	0.05	−0.04	0.05		−0.16	0.02	−0.06	−0.01				
	−0.72	−0.04	0.03		3.20	0.10	0.52					
E			−0.09	−0.14	−0.03	−0.09	0.03		−0.07	−0.03	−0.03	−0.02
	−0.66	0.18	0.40		0.17	0.27	−0.48		0.18	−0.14	0.23	

[a] *Chemical shift differences (^{1}H, top; ^{13}C, bottom) in ppm. Data for the wild-type GBS type III 14-repeating unit polysaccharide from Brisson et al. (1997). Brisson, J. R., Uhrinova, S., Woods, R. J., van der Zwan, M., Jarrell, H. C., Paoletti, L. C., et al. (1997). NMR and molecular dynamics studies of the conformational epitope of the type III group B Streptococcus capsular polysaccharide and derivatives. Biochemistry 36, 3278–3292. doi: 10.1021/bi961819l*

SUPPLEMENTARY TABLE S4. ^{1}H and ^{13}C NMR chemical shifts of the GBSVsia2,6 mutant CPS[a].

**Residue**		**1**		**2**	**3**		**4**	**5**	**6**		**7**	**8**	**9**		**CH_3**	**CO**
A	→4)-β-D-Glc-(1→	4.35		3.38	3.66		3.70	3.61	3.99	3.85						
		105.63		75.72	77.11		81.39	77.63	62.82							
B	→6)-β-D-Gal-(1→	4.45	(7.7)[b]	3.55	3.66		3.93	3.81	3.97	3.60						
		106.12		73.58	75.29		71.21	76.41	66.01							
C	→3,4)-β-D-Gal-(1→	4.53	(7.1)	3.77	3.85		4.26	3.81	3.88	3.84						
		105.81		73.31	83.49		78.08	78.36	62.82							
D	→4)-β-D-GlcNAc-(1→	4.58	(7.6)	3.76	3.77		3.67	3.59	3.96	3.87					2.10	
		103.45		57.89	75.04		83.17	77.06	63.13						25.53	176.67
E	β-D-Glc-(1→	4.64	(7.4)	3.19	3.51		3.36	3.43	3.93	3.73						
		107.15		76.41	78.52		72.81	78.59	63.83							
F	α -D-Glc-(1→	4.92		3.54	3.88		3.61	4.39	4.08	3.99						
		101.44		73.98[c]	73.93[c]		81.96	71.51	69.09							
G	α-D-Neu5Ac-(2→				2.68	1.68	3.67	3.78	3.71		3.56	3.88	3.87	3.65	2.04	
		175.98		103.06	42.84		70.94	54.72	75.31		71.32	74.48	65.78		24.86	177.66

[a] *Chemical shifts (^{1}H, top; ^{13}C, bottom) at 61°C in 33 mmol/L phosphate buffer pD 8.0 in D_2O in ppm referenced to internal DSS. ^{1}H chemical shifts were obtained from the 1D, COSY, TOCSY, or HSQC spectrum. ^{13}C chemical shifts were obtained from the 1D spectrum.*

[b] *Coupling constants ($J_{H\text{-}1\text{–}H\text{-}2}$) in parentheses.*

[c] *Tentative assignments.*
